# Walking the Talk: How to Identify Anti-Pluralist Parties

**DOI:** 10.1177/13540688231153092

**Published:** 2023-05-17

**Authors:** Juraj Medzihorsky, Staffan I. Lindberg

**Affiliations:** Durham University, Durham, UK; V-Dem Institute, University of Gothenburg, Göteborg, Sweden; V-Dem Institute, University of Gothenburg, Göteborg, Sweden

**Keywords:** anti-pluralism, party rhetoric, autocratization, populism, ideology

## Abstract

The recent increase of democratic declines around the world – “the third wave of autocratization” – has sparked a new generation of studies on the topic. Scholars tend to agree that the main threat to contemporary democracy arises from democratically elected rulers who gradually erode democratic norms. Is it possible to identify future autocratizers before they win power in elections? Linz (1978) and Levitsky and Ziblatt (2018) suggest that a lacking commitment to democratic norms reveals would-be autocratizers before they reach office. This article argues that the concept of anti-pluralism rather than populism or extreme ideology captures this. We use a new expert-coded data set on virtually all relevant political parties worldwide from 1970 to 2019 (V-Party) to create a new Anti-Pluralism Index (API) to provide the first systematic empirical test of this argument. We find substantial evidence validating that the API and Linz’s litmus-test indicators signal leaders and parties that will derail democracy if and when they come into power.

## Linz’s litmus test and the anti-pluralism index

Paradoxically, democracy’s contemporary enemies are elected leaders and parties that erode democratic rights and institutions (Bermeo 2016; Boese et al., 2021; Lührmann and Lindberg 2019; Svolik 2015). Once autocratization gets underway, democracies stand only about a 20% chance to avoid breakdown, with 36 of them breaking down since the 1990s (Boese et al., 2021). The key to democratic resilience that now preoccupies many in the academic and policy communities is therefore what prevents autocratization to begin with. A first step towards this goal is to answer the question: Can we identify the traits of political parties that erode democracy once in power?

Using a unique dataset on 1,943 political parties across 1,759 elections in 169 countries from 1970 to 2019 (Lührmann et al., 2020a), this article shows that we can identify such parties relatively well. The traits that signify would-be autocratizers are the four characteristics suggested in Linz’s (1978) canonical contribution: A rhetoric that is only “semi-loyal” to democratic norms, values, and institutions.^1^ We argue that “illiberalism” is a misleading term, conceptualize these four traits as anti-pluralist, and show how this differs from other suggestions such as populism (Müller 2017; Mudde and Kaltwasser 2013; Norris and Inglehart 2019) and ideology. We also aggregate the four indicators into the new Anti-Pluralism Index (API).∗ As an additional validation, the article demonstrates with the first ever systematic test how well the API and its four Linzian indicators identify seemingly democratic but will-be-autocratizing parties before they come into power.

Our approach follows a long tradition in comparative politics emphasizing that *political elite actors* rather than structural conditions decide the fate of democracy (e.g. Linz 1978; Bermeo 2003; Mainwaring and Perez-Linan 2013). Political parties are the key actors in a democratic system (Aldrich 2011). In Katz’s (1980: 1) classical insight, the “character of the parties in a political system is intimately related to the quality of its democracy”. Correspondingly, Ziblatt (2017) shows that it was the strategic decisions of (conservative) parties that sealed the fate of democracy in the Interwar years. Our focus here is on parties in the current period.

The article first discusses existing suggestions of which party/leader characteristics relate to autocratization and develops the argument for anti-pluralism. Second, we operationalize the Linzian indicators using the V-Party data set, aggregate them into the API, and analyze the validity of these measures. Third, as further validation of these “early warning signals” but also a substantive finding we demonstrate a strong relationship between the new indicators of party anti-pluralism and subsequent autocratization, concluding with a discussion of the findings’ implications for early warning systems and safeguards.

## Populism, ideology, or anti-pluralism?

Some 25 years after the Portuguese Carnation revolution started the “third wave of democratization” in 1974, Vladimir Putin rose to Prime Minister in President Yeltsin’s democratically elected administration on 16th August 1999 and then to president on 26th March 2000, winning 53% of the vote in relatively free and fair elections. The “third wave of autocratization” (Lührmann and Lindberg 2019) was getting underway. As a marker of what was going to come, Portugal’s democratization was initiated by a military coup, while Russia’s autocratization took off with a democratic election. Characteristically for recent and present autocratizations, democratically elected parties and their leaders emasculate democratic norms and institutions to curtail competition (e.g. Cassani and Tomini 2020; Diamond 2020; Haggard and Kaufman 2016; Lührmann and Lindberg 2019; Norris and Inglehart 2019; Plattner 2015). In contrast, most 20th century autocratizations originated in unconstitutional changes such as military coups, foreign interventions, and autogolpes (Bermeo 2016).

In the past 120 years, only about 20% of democracies that experienced autocratization onset survived (Boese et al., 2021). It is thus critical to identify the traits of political parties that erode democracy once they come into power. That would enable both an “early warning system” and studies of how such parties gain ground and reach power.

### Signifiers of political parties that derail democracy

We find three sets of arguments in the literature on the distinguishing characteristics of parties and leaders that derail democracy: populism, far-right or far-left ideologies, and “illiberalism” or as we suggest is preferable to label it: anti-pluralism. Although there are some conceptual overlaps between the three, we suggest that neither the core attributes of populism nor of extreme ideologies are the strongest identifiers of parties and leaders that undermine democracy once in power. Rather, we argue that lacking commitment to democratic norms as such – anti-pluralism – is the key sign of would-be autocratizers. Thus, we argue that the “illiberalism” label risks being misleading.

#### Populism

Many analysts equate populism with direct threats to democracy (e.g. Plattner 2010; Müller 2017; Galston 2017). Others see populism as a threat to democracy in its connection to corruption, the suppression of a critical civil society, and an exclusionary division of peoples into ‘us’ and ‘them’ (e.g. Galston 2018). Scholars usually agree on three core characteristics of populism: people-centrism, anti-elitism, and an antagonism between the “virtuous people” and the “corrupt elite” (e.g. Mudde and Kaltwasser 2013; Rooduijn 2014; Hawkins 2009).

By these traits alone, populism is not necessarily antidemocratic (see also Pappas 2016). Consequently, not all populists in power undermine democracy. Some researchers therefore distinguish between populist rhetoric and anti-democratic traits. For example, Norris and Inglehart (2019: 4, 7) distinguish between populism as an anti-elitist rhetoric, and authoritarianism as denying “liberal autonomy for the individual”. Similarly, Akkerman (2003) finds that only populist parties pushing for radical reform are a threat, while populists who respect institutional pluralism are not. Populist parties can be progressive, conservative, socialist, or authoritarian in the two-dimensional economic-GALTAN space (Akkerman and Rooduijn 2015; Norris and Inglehart 2019: 4).

In sum, the core of populism is arguably an anti-elitist and people-centric rhetoric (Hawkins 2009) that is neither theoretically nor empirically necessarily incongruent with commitment to democratic norms.

#### Ideology

Is an alternative suggestion for what threatens democracy (Mudde and Kaltwasser 2013; Akkerman 2003: 38). In reference to the classic left-right scale defined by economic policy, some argue that extreme left threatens democracy via its fervor for state interventionism that facilitates incumbent hegemony (Schamis 2006; Weyland 2013) citing cases of eroding democratic norms in countries such as Ecuador, Bolivia, and Venezuela (Levitsky and Roberts 2011: 399). Historically, conservative parties also used to oppose democracy, but we have not found empirical studies suggesting that current parties with far-right *economic* policies are likely to erode democracy.

With reference to the GALTAN dimension^2^ the threat to democracy is expected from ideologies of far-right traditionalists. For example, using a pooled cross-sectional design and data from 30 European countries from 1990 to 2012, Huber and Schimpf (2017) show that traditionalist right-wing parties have negative effects on minority rights, and that such parties are associated with propagation of violence and racism (Koopmans 1996).

Yet it seems that extreme ideologies in themselves do not automatically equate threat to democracy. With data on over 1,700 powerful political actors in 20 Latin American countries over a span of 66 years, Mainwaring and Perez-Linan (2013) show that privileging process over policy goals among powerful political actors is key: “competitive regimes are highly vulnerable to breakdown if the most powerful actors are indifferent to liberal democracy’s intrinsic value” (Mainwaring and Perez-Linan 2013: p.135). Recent experimental work shows that citizens who feel represented by the executive are more willing to delegate the president more authority even at the expense of democratic principles, and that the magnitude of the effect increases with partisan attachment (Graham and Svolik 2020; McCoy et al., 2020; Singer 2018; Svolik 2020). Hence, also ideologically relatively moderate parties can endanger democracy. The key is rather that when ideology beats adhering to process and norms, democracy is at risk. It thus seems to be less ideology at the extremes than prioritizing goals over democratic norms that is an important factor. Therefore, we turn to the third approach.

#### Anti-pluralism

The reasoning above leads us to the intuition that future autocratizers are signified by lacking commitment to democratic institutions, procedures, and norms. This should be fairly common-sense and follows insights by Linz (1978), Levitsky and Ziblatt (2018) and Müller (2012).

To capture this phenomenon, the term *illiberalism* recently gained prominence in part due perhaps to its use by Prime Minister Orbán in Hungary. Among others, scholars like Zakaria (1997) and Pappas (2016: 31) use “illiberalism” to denote a system of government that holds multiparty elections but does not protect basic liberties, and “illiberals” for the leaders seeking to derail democracy. However, these terms suffer from frequent misinterpretations. “Liberal” with its Latin root *lïberälis* has many meanings, from open-mindedness to broad-based education favoring independent thinking; state non-interventionism in the economy; social reforms; and designation for various political parties. As political actors like Orbán know, the many meanings open up for (mis)interpretations and disguising anti-democratic actions.

Instead, we suggest to anchor the analysis in that most definitions of democracy rest on the foundation of pluralism (Dahl 1971). As discussed for example by Sartori (1997), pluralism is a value system positing tolerance and respect for opposing views on the basis of mutual reciprocity. In Popper’s (1945: 293) words, pluralists “tolerate[s] all who are prepared to reciprocate, i.e. who are tolerant”. Pluralism requires consensus on the principle of reciprocal tolerance. This principle also informs the democratic norm that while the majority may rule, its legitimate course of actions is limited—they must respect the rights of minorities.

While some authors (Mudde and Kaltwasser 2013; Müller 2017; Norris and Inglehart 2019: 51) view pluralism as the opposite to populism, the latter is not necessarily anti-pluralistic. Anti-pluralism has four key characteristics in line with Linz’s (1978: 29) “litmus test” and Levitsky and Ziblatt’s (2018) four indicators that build on it. Drawing on these works, we argue that the first characteristic is *unwillingness to commit to the democratic process* as legal means for gaining power. Dahl (1971) formulated the minimum requirements in terms of institutional guarantees safeguarding true pluralism including orderly alternations. When parties do not commit to respect the institutions that regulate the means to access power, it signals anti-pluralism.

The second attribute is *denying the legitimacy of dissenting parties and opponents*, following directly from the principle of reciprocal tolerance on which pluralism rests. If political actors instead seek to delegitimize, severely personally attack, or demonize their opponents, it indicates lacking commitment to pluralism (Levitsky and Ziblatt 2018: 23–24). The only exception is the legitimate denial of legal existence to parties that do not accept the principle of pluralism (see e.g. Popper (1945: 130) in his work on the paradox of freedom).

The third key feature is *toleration or endorsement of the use of political violence*. Pluralism is predicated on the principle that the law protects citizens’ civil liberties from arbitrary violation by the state and elected representatives (Merkel 2004: 39). Thus, a key signal of weak commitment to pluralism is supporting that the will of the majority should be implemented even if doing so would violate the civil liberties of opponents. Consequently, toleration or endorsement of the use of political violence should be a signal of an anti-pluralist party or leader (Levitsky and Ziblatt 2018: 23–24).

Finally, *indications that a party and its leaders could consider curtailing the civil liberties of minority groups* characterize anti-pluralists. Dahl (1971) rightly emphasize that democracy requires a plurality of opinions that can be expressed freely, including by minorities. Civil liberties thus enable and are fundamental prerequisites for pluralism.

In sum, anti-pluralist parties lack commitment to *i) the democratic process as the legal means of gaining and losing power; ii) the legitimacy of political opponents; iii) peaceful resolution of disagreements and rejection of political violence; and iv) unequivocal support for civil liberties of minorities.*

Following the literature, we submit that political parties registering weak or no commitment in these areas are likely autocratizers if and when they are elected to form a government. Until now, however, we lacked the cross-national data needed to test this proposition.

## Measuring the markers of anti-pluralists

The reasoning above coheres with Linz’s (1978: 29) “litmus test” of what characterizes political actors semi- or even disloyal to the democratic system and Levitsky and Ziblatt (2018)’s version (see Table 1).^3^

**Table 1.** Indicators of anti-pluralist political actors.

Linz (1978)	Levitsky and Ziblatt (2018)	Lührmann et al. (2020a)
Unwillingness to publicly commit to legal means for gaining power	Expresses willingness/need to violate the constitution; Expresses sympathy for non-constitutional means of accessing power; Attempts to undermine the legitimacy of election	Low commitment to the democratic process
Denial of the legitimacy of democratic political parties to participate in political processes	Describes rivals as subversive, criminal, or foreign agents; Claims that rivals constitute existential threat	Demonization of political opponents
No rejection of the use of force; Willingness to ask for the armed forces	Has ties to armed gangs or militias; Sponsors or encourages mob attacks on opponents; Endorses or praises political violence	Encouragement of political violence
Curtailment of the civil liberties of democratic parties’ leaders and supporters	Supports laws or policies restricting civil liberties; Threatens to take legal action against critics; Praises repression	Disrespect for fundamental minority rights

Source: Linz (1978); Levitsky and Ziblatt (2018); Lührmann et al. (2020a)

When considering suitable indicators we must recognize that anti-pluralist parties typically seek to shore up their credentials as “regular democratic parties” in official party documents. For example, data from the Manifesto Project (Volkens et al., 2019) show that such parties routinely pay lip service to democracy: The German far-right party AfD scores high (4.08) and the center-right party CDU low (0.74) on their measure of favorable mentions of democracy in 2017. Thus, party documents alone are not adequate.

Rhetoric in speeches and other events can be more authentic and revealing (Maerz and Schneider 2021). Maerz and Schneider (2019) compared 4,740 speeches from heads of government in 27 countries between 1999 and 2019, and found that leaders in autocratic countries use a substantially less democratic style of rhetoric than leaders of democratic countries. Linz (1978) found that political parties that later seek to derail democracy are typically explicit with their true anti-pluralism also before they assume politically powerful positions. Unfortunately, the availability of party leaders’ recorded public speeches is unequal and biased towards developed countries with large media infrastructures, as well as towards the present. We therefore turn to measures on anti-pluralism in the V-Party data set to operationalize the Linzian indicators (Lührmann et al., 2020a):

• **Low commitment to the democratic process (v2paplur)**: “Prior to this election, to what extent was the leadership of this political party clearly committed to free and fair elections with multiple parties, freedom of speech, media, assembly and association?”
• **Demonization of political opponents (v2paopresp)**: “Prior to this election, have leaders of this party used severe personal attacks or tactics of demonization against their opponents?”
• **Encouragement of political violence (v2paviol)**: “To what extent does the leadership of this party explicitly discourage the use of violence against domestic political opponents?”^4^
• **Disrespect for fundamental minority rights (v2paminor)**: “According to the leadership of this party, how often should the will of the majority be implemented even if doing so would violate the rights of minorities?”^5^

The V-Party dataset is based on 665 country experts assessing the identity of all political parties with a vote share of more than 5% in a legislative election between 1970 and 2019, across 169 countries (Lührmann et al., 2020a). The data covers 1,955 political parties across 1,560 elections—or in total 6,330 party-election year units. Typically, more than four experts rated each country-year-question combination.^6^ All items are measured on a five-point ordinal scale, and aggregated correcting for possible between-expert differences in scale use using V-Dem’s IRT model (Marquardt and Pemstein 2018; Marquardt et al., 2019; Pemstein et al., 2020). Validation of the V-Dem approach of expert-coding has been extensive and positive. When constructing the API, we use the MM versions of the four Linzian indicators, while when using them directly, we take their OSP versions (Coppedge et al., 2020) linearly rescaled onto [0,1] so that higher values indicate higher levels of anti-pluralism. For details see the SM.

### Index aggregation

We aggregate the four indicators into the new Anti-Pluralism Index (API) recognizing that the violations they tap into are not equally severe. In particular, an explicit denial of democratic institutions such as elections is a more severe rejection of pluralism than harsh language towards opponents. Based on this reasoning, we compute the index as a transformed weighted average of the input indicators using the following formula:

(1) $$\begin{array}{rl} v2xpa\_rival_{i}=1-\phi (\frac{1}{4.5}( & 0.5\times v2paopresp_{i}\\ & +\;2\times v2paplur_{i}\\ & +\;v2paviol_{i}\\ & +\;v2paminor_{i})), \end{array}$$

where *i* indexes observations, Φ is the standard normal cumulative density function, and the four indicators are Demonizing opponents (v2paopresp), Low commitment to democratic processes (v2paplur), Disrespect for fundamental minority rights (v2paminor), and Encouragement of political violence (v2paviol). Supplemental Figure A in the SM shows the joint distributions of the API and its components.

### Data validation

**Figure 1.**
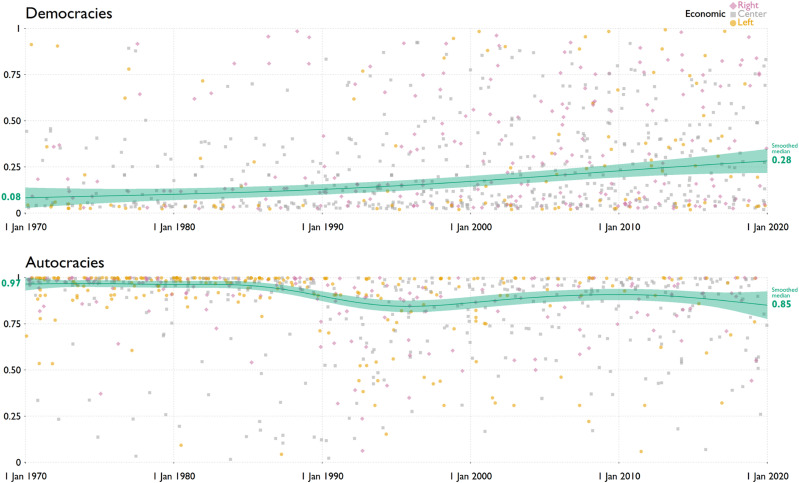
Anti-Pluralism Index (API) of parties that gained or retained the Head of Government post in national elections. Smoothed medians and 95% regions estimated under quantile GAMs with GP smooths. Elections split by regime (v2x_regime): autocracies {0,1}, democracies {2,3}. Color by economic left-right on the OSP scale: left [0,2], center (2,4], right (4,6]. Adapted from Lührmann et al. (2020b).

Since no other measure has yet captured the anti-pluralist traits of political parties, we assess *convergent validity* by comparing the values on the API of ruling parties in democracies (top of Figure 1) with the one in autocracies (bottom).

Reassuringly, this shows stark differences. The smoothed median score in 2019 was 0.28 in democracies and 0.85 in autocracies. The median governing party in democracies has become more anti-pluralist in recent decades, which is congruent with research showing that contemporary threats to democracy typically come from within the government (Bermeo 2016). The median governing party in autocracies has become somewhat less anti-pluralist in the same period, reflecting the mimicking of multi-party elections in most autocracies (Schedler 2002). This finding thus supports the not only the convergence but also the content validity of the new index.

**Figure 2.**
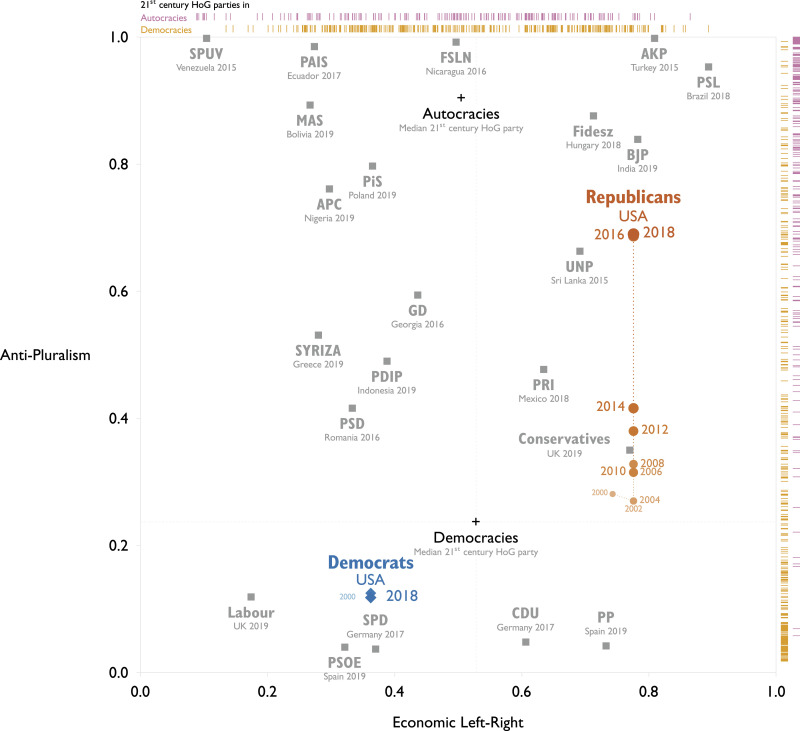
The movements of the two major US parties on the Anti-Pluralism Index and economic left-right since 2000.

To further assess the *content validity* of the API, we explore some relevant cases. Figure 2 highlights the US Republican and Democratic parties on two dimensions: anti-pluralism and left-right positioning on economic policy, with other parties positioning at the last election in relief. The Republican party has not changed its left-right placement but moved strongly in an anti-pluralist direction reaching an API score of 0.69 in 2018. This reflects that Trump in his 2016 presidential campaign made personal, demonizing attacks on political opponents leading to a high score of 0.86 on the demonization indicator (see Table 2).^7^ He also condoned violence towards his political opponents, saying about Clinton that *“If she gets to pick her judges, nothing you can do, folks […] Although—the second amendment people—maybe there is”*,^8^ and towards protesters at his rallies. This is reflected in the score for the encouragement of violence (0.35, Table 2).

**Table 2.** Scores of selected parties on the Anti-Pluralism Index and its component indicators. Indicators have reversed scales to range from 0 (pluralist) to 1 (anti-pluralist).

Party	Country	Election	Anti-pluralism index	API component indicators			
				Demo-cratic process	Demo-nization	Disrespect minorities	Encourage-ment of violence
AKP	Turkey	2018	1	0.96	0.99	0.93	0.95
PSL	Brazil	2018	0.95	0.53	0.91	0.88	0.53
Fidesz	Hungary	2018	0.88	0.41	0.96	0.79	0.38
PiS	Poland	2019	0.80	0.35	0.91	0.71	0.37
Republicans	USA	2016	0.69	0.28	0.86	0.61	0.36
SYRIZA	Greece	2015	0.53	0.18	0.60	0.49	0.52
Conservatives	UK	2019	0.35	0.09	0.87	0.82	0.04
Democrats	USA	2016	0.12	0.13	0.37	0.31	0.02
CDU	Germany	2017	0.05	0.05	0.15	0.27	0.04

Already by 2016, the rhetoric of GOP’s leaders was closer to autocratic ruling parties such as the Turkish AKP (1.0) and Hungarian Fidesz (0.88) than to typical center-right governing parties in democracies such as the Conservatives in the UK (0.35) or CDU in Germany (0.05).^9^ The same holds for Bolsonaro (PSL, Brazil) whose rhetoric during the 2018 presidential campaign frequently demonized his opponents and promoted violence (Hunter and Power 2019), reflected in PSL’s API of 0.95. Likewise, PiS, ruling in Poland since 2015, scored 0.80 on the API by the 2019 elections, reflecting for instance that the government-controlled media attacked and demonized PiS’ opponents and accused them of threatening traditional Polish values (Markowski 2020).

The Greek SYRIZA is on an intermediate level of anti-pluralism (0.53 ahead of the 2015 election). SYRIZA accused opponents of being subservient to foreign powers and the international banking system (Aslanidis and Rovira Kaltwasser 2016) and correspondingly did not score well in opponent demonization (0.60). In comparison, the leadership of Angela Merkel’s CDU made no known anti-pluralist statements or behaviors, which corresponds to a low API score of 0.05 in 2017.

### Validating the relationship between anti-pluralism as indicated by the API and autocratization

Table 3 shows that when anti-pluralist parties gained power in democracies, 29% autocratized in the year after they won (or defended) office (see Group IV). After pluralists won office, only 6% autocratized (Group II) while most remained democratic (Group I), an outcome that was much less likely after the election of anti-pluralists (Group III). Meanwhile the result for Group I is not surprising.

**Table 3.** Anti-pluralists winning elections in democracies and autocratization (t + 1), 1970–2018.

	No autocratization	Autocratization	
Pluralists in power	542 (94%)	36 (6%)	578 (100%)
	I: Democratic stability with pluralists governing	II: Autocratization with pluralists governing	
Anti-pluralists in power	137 (71%)	56 (29%)	193 (100%)
	III: Democratic stability with anti-pluralists governing	IV: Autocratization with anti-pluralists governing	
All	679 (88%)	92 (12%)	771

**Figure 3.**
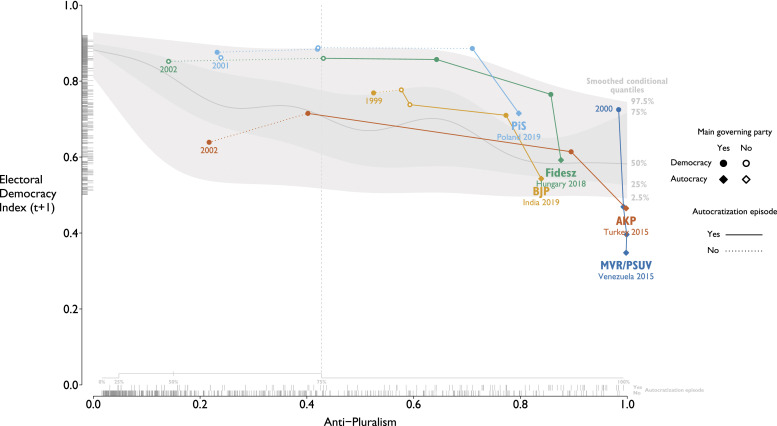
Anti-pluralism of governing parties in election years in democracies and V-Dem’s Electoral Democracy Index in the first post-election year (*t* + 1). Smoothed conditional quantiles estimated under quantile GAMs with GP smooths. Some of the highlighted party trajectories include elections under non-demoratic regimes.

Figure 3 shows the relationship between the API of ruling parties (*x*-axis) and the level of electoral democracy (*y*-axis): the more anti-pluralist ruling parties become, the lower the level of democracy. When political parties move to the right of the dotted vertical line (at the API value of 0.43), they are considered anti-pluralists. Trajectories of selected *Group IV* parties are highlighted. The Polish Law and Justice Party (PiS), the Hungarian Fidesz, and the Turkish Justice and Development Party (AKP) all started with scores in the pluralist part of the spectrum, while The Indian Bharatiya Janata Party (BJP) exhibited some level of anti-pluralism already in 1999. All four parties have become increasingly anti-pluralist and autocratization has followed.

The AKP increased its anti-pluralist traits between every election since its foundation. When Erdoğan came to power after the 2002 election, he had promised reforms that would enhance the separation of powers, the independence of the judiciary, increase the freedom of press, and strengthen the rule of law. However, Erdoğan have instead been cracking down on protesters (Taspinar 2014), orchestrating prosecutions of political opponents, and describing the separation of powers as “an obstacle” that he would overcome by hollowing out the judiciary (Karaveli 2016).

Polish PiS has gone from an API of 0.23 in 2005 when it first gained power, to 0.71 when it returned to power in 2015. This reflects its increasing nationalism, disrespect of minority rights, and demonizing of opponents (Harper 2010: 24). Additionally, PiS has undermined the independence of the judiciary, checks and balances, as well as freedom of expression (Markowski 2020). Meanwhile Poland’s EDI score declined from 0.89 in 2014 to 0.69 in 2019.

Hungary was an electoral democracy in 2010 when Fidesz won the elections and returned to power. The Orbán government has since placed constrains on civil society, restricted freedom of expression as well as academic freedom,^10^ and blatantly demonized the opposition.^11^ A substantial decline on the EDI followed, from 0.81 in 2010 to 0.49 by 2018.

The MVR/PSUV in Venezuela scored high on anti-pluralism (0.95) already in 1998 when Chávez won his first election. Venezuela then embarked on a drastic autocratization episode with its EDI score dropping from 0.72 in 1999 to 0.33 by 2015. After PSUV lost the 2015 legislative elections, Maduro (Chávez’s successor) stripped the parliament of power and designated the Supreme Court to take over the functions of the National Assembly (Alarcon et al., 2016; Corrales 2020).

Democracy has deteriorated since the BJP led by Narendra Modi became India’s governing party in 2014. India’s EDI declined from 0.69 then to 0.51 in 2019. A series of policies have diminished freedom of expression and academic freedom, and repression of civil society has increased along with persistent discrimination against Muslims (Ganguly 2020; Varshney 2019). This is reflected in BJP’s API of 0.84 in 2019.

However, not all countries autocratize with anti-pluralists in office (*Group III*). In 137 cases, autocratization did not start in the year after an anti-pluralist came to power (lower rug on the right of Figure 3). The median API in the group (0.68) is lower than in the group where anti-pluralism is followed by autocratization (0.77). This may indicate that a certain threshold is required for autocratization to start, which is an avenue for future research. Research indicates that stronger parliamentary and judicial oversight can help to prevent autocratization (Boese et al. 2021). A moderately anti-pluralist party that did not substantially erode democracy was Forza Italia with an anti-pluralism score of 0.72 in 2001. Silvio Berlusconi then governed Italy in 2001-2006 and 2008-2011, and while he engaged in anti-pluralist rhetoric, the Italian institutions withstood the pressure and remained more or less intact (Verbeek and Zaslove 2016).

A few elections (36) were followed by autocratization even though a pluralist party was in office (*Group II*). For example, Citizens for European Development of Bulgaria (GERB) scored 0.13 on the API when gaining power in 2009. Yet, the quality of democratic institutions declined substantially from 2009 (EDI 0.72) to 2019 (EDI 0.59). GERB pursued an agenda of state capture putting loyal individuals in charge of media outlets, the Supreme Justice Council, and anti-corruption agencies (Ganev 2018). A less pronounced autocratization process occurred in Chile under pluralist leadership from 2011 to 2019,^12^ and in Israel (2010–2019).^13^ In other cases, the pluralist party leader wining the elections was removed from office under dubious circumstances as with President Dilma Rousseff in Brazil in 2016 (Chalhoub et al., 2017). Finally, some cases in this group registered only minor democratic declines under pluralist rule but more severe declines later under anti-pluralist rule, for instance in Hungary in 2007 and the United States in 2015.

We hope that the above conveys the construct and convergent validity of the API and its four indicators by that: (i) developments on the API correspond to real-life events; (ii) it captures anti-pluralist traits of ruling parties before they begin eroding democracy; (iii) the resolved data show developments over time; (iv) the measures of autocratization and anti-pluralist rhetoric capture distinct concepts.

## Does anti-pluralism signify would-be autocratizers?

Building on Linz (1978), the hypothesis is that anti-pluralism is an *indication*. All else equal, anti-pluralism should signal increased risk that parties undermine democracy if they assume power. The Anti-Pluralism Index and its constituent indicators allow the first global empirical test of this.

Alternatively, one may contend that anti-pluralistic parties should be expected to i) try conceal their lack of commitment to democracy before obtaining power due to the appeal of moderate voters; ii) fail to autocratize if they end up in coalition governments; iii) sometimes not be able to autocratize even if they wish to; and other parties may pretend to be more anti-pluralist than they are in times when popular sentiments tend towards anti-pluralism. These are definitive possibilities but in all cases would lead in the opposite direction to the main Linzian hypothesis. Thus, the analyses below are both an exercise of hypothesis evaluation as well as further *construct validation* (Adcock and Collier 2001).

To analyze the relationship between the characteristics of political parties’ rhetoric before, and the behavior of political leaders after (re-)assuming office, we focus on the relevant subgroup of 771 cases defined above: parties in democracies that head government after a given election.^14^

Our dependent variable equals 1 if the country is undergoing substantial autocratization in the year after the elections and 0 if it is not. Such autocratization episodes represent a decline in V-Dem’s Electoral Democracy Index (EDI) of more than 10% of the index value during one year or over a connected time period (Maerz et al., 2021). Operationalizing autocratization in this way is superior to a simple year-to-year change as it allows us to capture substantial and gradual processes of autocratization, while at the same time not registering year-to-year fluctuations that may be measurement noise.

If we find a positive relationship as expected, it would both be an additional and stronger validation of the API and the four Linzian indicators, as well as a substantive contribution to the literature.

### Regression design

To capture possible nonlinearities, we use Generalized Additive Models (GAM) under which party characteristics as well as some additional covariates are included via Gaussian Process (GP) smooths (see e.g. Hastie and Tibshirani 1990). Under a binary *y*-variable, the Gaussian GAM is a more flexible counterpart of the popular Linear Probability Model (see e.g. Angrist and Pischke 2008), and the smooths may be interpreted analogically to slopes under the LPM.

Following the arguments that populism and extreme ideology make parties threaten democracy, we include their measures from the V-Party data. The populism measure rests on a conceptualization that combines parties’ anti-elitist rhetoric and “glorification of the ordinary people and identify themselves as part of them” (Lührmann et al., 2020a: 26).^15^ Second, we evaluate the conventional economic left-right dimension of the party system.^16^ Third, we capture the distinction between culturally progressive and conservative parties with a socio-cultural index.^17^ For more details on these measures see the SM. When used in regressions, all party characteristics are scaled from 0 (left/non-populist) to 1 (right/populist).

#### Contextual covariates

To adjust for the socio-economic and political context in the pre-election year (*t* − 1) we include additional covariates. First, since the likelihood of autocratization as well as the election of anti-pluralist parties should vary with the level of horizontal constraints on the executive, we include V-Dem’s Liberal Component Index (LCI) (Coppedge et al., 2020). It captures both legislative and judicial constraints on the executive, and the rule of law. Democracies are also said to be more likely to break down if they have a presidential system (Linz 1978; Svolik 2008). Therefore, we adjust for presidential systems.^18^

Third, both lower level of economic development (Przeworski et al., 2000) and economic crises (Bernhard et al., 2001) are associated with democratic breakdowns. Therefore, we adjust for GDP/capita (natural logarithm) and GDP growth/capita using data from the Maddison project (Bolt et al., 2018).^19^ An equal distribution of resources reduces the likelihood of autocratization (e.g. Haggard and Kaufman 2016). Therefore, we account for inequality with V-Dem’s Equal Distribution of Resources Index.

Finally, it seems plausible that autocratization becomes more likely in a global climate of many reverse trends and less likely in a context of democratization (Lührmann and Lindberg 2019). Therefore, we adjust for the share of countries going through autocratization and democratization episodes each year.^20^ For similar reasons, we adjust for the average regional EDI, always excluding the country of observation from the average (Coppedge et al., 2020). We also include year to account for temporal effects and allow for non-linear relationship with a GP smooth.

### Results

**Figure 4.**
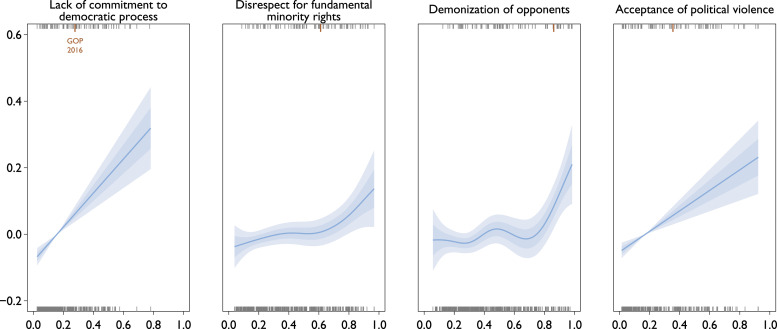
Modeled Probability of Autocratization in the Year after Election (Indicator-level). Partial effects under Gaussian GAMs with GP smooths (*N* = 771). Each model includes the plotted index and adjusts for the same set of contextual coviariates (see above). Upper rugs show observations with autocratization at *t* + 1, lower rugs observations without it. 2016 US Republicans highlighted with a longer orange tick. Shaded ± 1SE and ± 2SE regions. Model summaries reported in Supplemental Table B in the SM.

Starting with the Linzian indicators, Figure 4 presents estimated conditional relationships between autocratization and the four components of the API. Each estimate comes from a separate model that includes all the contextual covariates described above. The *y*-axis shows the expected change in the probability of autocratization and the *x*-axis the values of the component. We report additional model specifications in the SM, all of which give substantively similar estimates unless noted otherwise here.

Weaker commitment to the democratic process is associated with a substantially greater probability of autocratization. This relationship is linear, and substantively significant starting already at relatively minor deviations from the democratic baseline (“[t]he party leadership was fully committed to free and fair, multi-party elections, freedom of speech, media, assembly and association”). We find a similar relationship to autocratization for acceptance of political violence.^21^ Disrespect for minority rights and demonization of opponents have somewhat similar, but non-linear and weaker relationships to autocratization. And, the relationships considerably weaken if the other three party attributes are included in the model specification (see Supplemental Table J in the SM).

What is the substantive implication of these findings? For the litmus test to work, what matters most are pairwise associations of each characteristic with autocratization. We find that for all four characteristics even conditioning on contextual covariates. However, when considering the four at the same time, it seems that lacking commitment to democratic process and accepting political violence are the most informative ones (Supplemental Figure I in the SM), but extreme levels of opponent demonization also associate strongly with higher autocratization rates.

Another perspective is to consider the capacity to predict autocratization. We quantify it by Area Under the receiver operator Curve (AUC), which has a straightforward interpretation as the proportion of all possible {*y*_*k*_ = 0, *y*_*k*′_ = 1} observation pairs in which the latter has a larger predicted value (*p*_*k*_ < *p*_*k*′_). We estimate AUC with leave-pair-out cross validation (Airola et al., 2009), by randomly sampling 10 thousand {0, 1} observation pairs and obtaining the predictions for them under the model re-estimated without the pair. A detailed summary features in Supplemental Table A in the SM. Lack of commitment to democratic process and acceptance of political violence already on their own predict fairly well, with AUCs of 0.77, and adding all contextual covariates increases the AUCs only somewhat, to 0.83.

**Figure 5.**
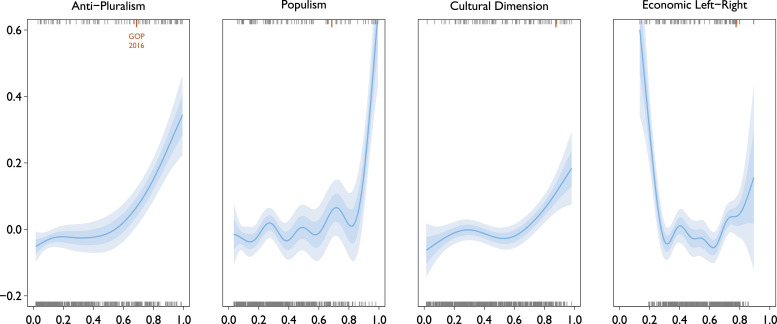
Modeled Probability of Autocratization in the Year after Election (Index-level) Partial effects under four Gaussian GAMs (*N* = 771). Each model includes the plotted index and adjusts for the same set of covariates. Upper rugs show observations with autocratization at *t* + 1, lower rugs observations without it. 2016 US Republicans highlighted with a longer orange tick. Shaded ±1SE and ±2SE regions. Model summaries reported in Supplemental Table C in the SM.

Figure 5 shows the estimated relationships with autocratization of the API and the alternative party characteristics under models that include socio-economic and political covariates. A greater level of anti-pluralism has a non-linear but strong association with autocratization. This relationship is substantively significant at high levels of anti-pluralism. For populism some relationship to autocratization can be seen; but only at *extremely* high levels of populism, for which there are only few observations. For culturally conservative parties we find a positive relationship to autocratization, but somewhat weaker and also only at very high levels of cultural conservatism. In terms of economic party positions only very extreme far-left associates with a clearly greater probability of autocratization and again based on a very small number of observations.

Out of the four, the API predicts autocratization the best, as captured by LPOCV-AUC (reported in detail in Supplemental Table A in the SM). Without covariates, the API model achieved an AUC of 0.79, while the populism model placed second with 0.66. With contextual covariates included, the API model is not outdone, with an AUC of 0.83. In short, the API on its own predicts autocratization nearly as well as any of the other indexes combined with contextual covariates.

To assess the sensitivity of our findings, we re-analysed our data under alternative model specifications and operationalizations of the autocratization variable. In the SM, we report several additional analyses:

• We re-estimate the models above for new incumbents only. Supplemental Figures O and P and Tables H and I in the SM summarize the estimates.
• We estimate the Gaussian GAMs in Figures 4 and 5 using two alternative operationalizations of the autocratization variable, namely autocratization episode at the second and at the third post-election year. Supplemental Figures I, J, K, and L report the estimates. All lead to the same substantive conclusions as the main analysis above.
• We reanalyse the data with Binomial-probit GLMs. Tables D and E, report the estimates. Again, these support the same substantive findings.
• Finally, we operationalize the autocratization variable with changes in the Revised Polity (Marshall et al., 2016) score between the last pre-election year and the first post-election year, and analyse the data with Gaussian linear regressions (OLS). Tables F and G report the estimates. For the party variables of interest, the point estimates are largely of the same direction and relative magnitude as in the main analysis. However, their associated standard errors are relatively large. This does not surprise as this operationalization measures incumbent-led autocratization at lower precision than our main approach.^22^

## Conclusions

What characterizes the parties and leaders that lead autocratization processes once in power? They lack commitment to democratic norms and processes, encourage violence, and demonize opponents. This answer might seem trivial to some given Linz’s (1978) litmus test and Levitsky and Ziblatt’s (2018) list of early-warning indicators. However, this article first disentangles the concept of anti-pluralism from notions of populism and ideology, thus extracting the operative elements that characterize parties that threaten present democracies. The article then provides an operationalization of these, details new unique indicators for the four critical aspects, and present the new API.

Our empirical analysis is the first to show quantitatively in a global sample that parties characterized by scoring high on the API and the four indicators are indeed “walking the talk” and their anti-pluralist rhetoric before they govern should be taken seriously. These findings are both validating the Linzian litmus test idea along with our operationalization in four indicators and the API, but are also a substantive contribution to the literature.

The tests presented are possible due to new data from the V-Party data set on the anti-pluralist traits of all 771 parties that won power in democracies between 1970–2018 (Lührmann et al., 2020a) and new data identifying autocratization (Maerz et al., 2021). The fine-grained expert-coded data allows us to differentiate between governments that autocratize after campaigning with an anti-pluralist agenda, and those that do not. As the findings in this article show, when a candidate for executive office *is* identifiable as anti-pluralist before the election, autocratization is more likely once they assume office. Although not all autocratizers provide such warning, most do. To safeguard liberal democracy, it is important to be alert for these early warning signals.

This study points at what kind of rhetoric and behavior provide such signals. Future research should examine both the factors that enable anti-pluralists to reach power, and constraining factors that might avert democratic breakdown even though an anti-pluralist is in power.

## Supplemental Material

Supplemental Material - Walking the Talk: How to Identify Anti-Pluralist Parties Supplemental Material for Walking the Talk: How to Identify Anti-Pluralist Parties by Juraj Medzihorsky and Staffan I Lindberg in Party Politics

## Notes

1. Based on this argument, Levitsky and Ziblatt (2018) laterrephrased them as a list of early-warning indicators for potential anti-democratic behavior.

2. Green, Alternative, and Libertarian parties are located on the progressive side, while Traditional, Authoritarian, and Nationalist parties are on the other side of the spectrum (Bakker et al., 2015; Hooghe et al., 2002; Marks et al., 2006; Rohrschneider and Whitefield 2009)

3. Müller (2012) suggests a similar set of characteristics but with a slightly more narrow scope.

4. Coders were shown the following clarification: “ ‘Domestic political opponents’ refers to all political opponents, with the exception of those who are engaged in an armed conflict with the state. They may be other political parties or other political groups and movements.”

5. Coder were shown the following clarification: “This concerns the rights enshrined in the Universal Declaration of Human Rights, which apply to everyone ‘without distinction of any kind, such as race, colour, sex, language, religion, political or other opinion, national or social origin, property, birth or other status.’ The declaration protects—among others—freedom of speech, property, religion, peaceful assembly and association.”

6. Mean = 4.55; median (p50) = 4; p25 = 3; p10 = 2.

7.
https://www.theguardian.com/us-news/2016/may/25/donald-trump-crooked-hillary-clinton-nickname-ignore-fight

https://www.theguardian.com/us-news/video/2016/aug/12/donald-trump-hillary-clinton-day-wrong-florida-video

https://abcnews.go.com/Politics/crooked-hillary-marco-donald-trumps-nicknames/story?id=39035114

8.
https://www.theguardian.com/us-news/2016/aug/09/trump-gun-owners-clinton-judges-second-amendment

9. In 2018 the AKP ran as part of the People’s Alliance.

10.
https://www.ceu.edu/istandwithceu/timeline-events

11.
https://www.nytimes.com/2018/04/07/world/europe/hungary-viktor-orban-election.html

12.
https://rsf.org/en/news/rsf-alarmedescalating-violence-against-reporterschile

13.
https://foreignpolicy.com/2010/03/10/civil-society-and-human-rights-in-israel-and-elsewhere/

14. To identify which party is in power we use an updated version of the variable v2pagovsup from (Lührmann et al., 2020a) and for democracies the variable v2x_regime in the year before elections from Coppedge et al. (2020).

15. The populism index (v2xpa_popul) is computed as theharmonic mean of rescaled anti-elitism (v2paanteli_osp) andpeople-centrism (v2papoeple_osp) measures, to reflect thetheory that populism requires both elements..

16. Experts were asked to “locate the party in terms of its overallideological stance on economic issues” on a 0 (far-left) to 6 (far right) scale. They were shown the following clarification:“Parties on the economic left want government to play anactive role in the economy. This includes higher taxes, more regulation and government spending and a more genereous welfare state. Parties on the economic right emphasize a reduced economic role for government: privatization, lower taxes, less regulation, less government spending, and a leaner welfare state”. (Lührmann et al., 2020a)

17. The index aggregates measures on immigration (v2paimmig), LGBT Social equality (v2palgbt), Cultural superiority (v2paculsup), religious principles (v2parelig), and workingwomen (v2pawomlab).

18. V-Dem data captures if the chief executive is unitary (v2exhoshog=1) and directly elected by the population (v2expathhs=7).

19. As included in Coppedge et al. (2020).

20. We created this measure by dividing the number of autocratization or democratization episodes by the number of countries per year (Maerz et al., 2021).

21. This association weakens some if the other three party attributes (populism, cultural dimension and economic left-right) are included in the model specification (see Supplemental Figure J in the SM).

22. It not only measures autocratization, but also democratization and captures minor fluctuations in democracy ratings.
