# Supplementary material for: Walking the Talk: How to Identify Anti-Pluralist Parties
Source: Party Politics. 2023 May 17;30(3):420–34. doi: 10.1177/13540688231153092 (PMC11069453; doi:10.1177/13540688231153092)
Supplement: Supplemental Material - Walking the Talk: How to Identify Anti-Pluralist Parties [file sj-pdf-1-ppq-10.1177_13540688231153092.pdf]

Supplemental material

for 'Walking the Talk: How to Identify Anti-pluralist Parties'

Expert survey items

This is the original question wording from the V-Party codebook.[23] Note that in this paper, we have flipped the scales of all four indicators, so that higher values signal higher levels of anti-pluralism.

Political opponents (v2paopresp)

Prior to this election, have leaders of this party use severe personal attacks or tactics of demonization against their opponents?

Clarification: Severe personal attacks and demonization includes dehumanizing opponents or describing them as an existential threat or as subversive, criminal or foreign agents.

0. Always. Party leaders always used severe personal attacks or tactics of demonization against their opponents.
1. Usually. Party leaders usually used severe personal attacks or tactics of demonization against their opponents.
2. About half of the time. Party leaders sometimes used severe personal attacks or tactics of demonization against their opponents.
3. Usually not. Party leaders usually did not use severe personal attacks or tactics of demonization against their opponents.
4. Never. Party leaders never used severe personal attacks or tactics of demonization against their opponents.

Democratic process (v2paplur)

Prior to this election, to what extent was the leadership of this political party clearly committed to free and fair elections with multiple parties, freedom of speech, media, assembly and association?

Clarification: Party leaders show no commitment to such principles if they openly support an autocratic form of government without elections or freedom of speech, assembly and association (e.g. theocracy; single-party rule; revolutionary regime). Party leaders show a full commitment to key democratic principles if they unambiguously support freedom of speech, media, assembly and association and pledge to accept defeat in free and fair elections.

0. Not at all committed. The party leadership was not at all committed to free and fair, multi-party elections, freedom of speech, media, assembly and association.
1. Not committed. The party leadership was not committed to free and fair, multi-party elections, freedom of speech, media, assembly and association.
2. Weakly committed. The party leadership was weakly committed to free and fair, multi-party elections, freedom of speech, media, assembly and association.
3. Committed. The party leadership was committed to free and fair, multi-party elections, freedom of speech, media, assembly and association.
4. Fully committed. The party leadership was fully committed to free and fair, multi-party elections, freedom of speech, media, assembly and association.

Minority rights (v2paminor)

According to the leadership of this party, how often should the will of the majority be implemented even if doing so would violate the rights of minorities?

Clarification: This concerns the rights enshrined in the Universal Declaration of Human Rights, which apply to everyone "without distinction of any kind, such as race, colour, sex, language, religion, political or other opinion, national or social origin, property, birth or other status." The declaration protects - among others - freedom of speech, property, religion, peaceful assembly and association.

0. Always. The leadership of this party argues that the will of the majority should always determine policy even if such policy violates minority rights.
1. Usually. The leadership of this party argues that the will of the majority should usually determine policy even if such policy violates minority rights.
2. Half of the time. The leadership of this party argues that the will of the majority should about half of the time determine policy even if such policy violate minority rights.
3. Usually not. The leadership of this party argues that the will of the majority should usually not determine policy if such policy violates minority rights.
4. Never. The leadership of this party argues that the will of the majority should never determine policy if such policy violates minority rights.

Rejection of political violence (v2paviol)

To what extent does the leadership of this party explicitly discourage the use of violence against domestic political opponents?

Clarification: "Domestic political opponents" refers to all political opponents, with the exception of those who are engaged in an armed conflict with the state. They may

be other political parties or other political groups and movements.

0. Encourages. Leaders of this party often encourage the use of violence against domestic political opponents.
1. Sometimes encourages. Leaders of this party sometimes encourage the use of violence against domestic political opponents and generally refrain from discouraging it.
2. Discourages about half of the time. Leaders of this party occasionally discourage the use of violence against domestic political opponents, and do not encourage it.
3. Generally discourages. Leaders of this party often discourage the use of violence against its domestic political opponents.
4. Consistently discourages. Leaders of this party consistently reject the use of violence against its domestic political opponents.

Data validation

Additional visualizations of anti-pluralist characteristics

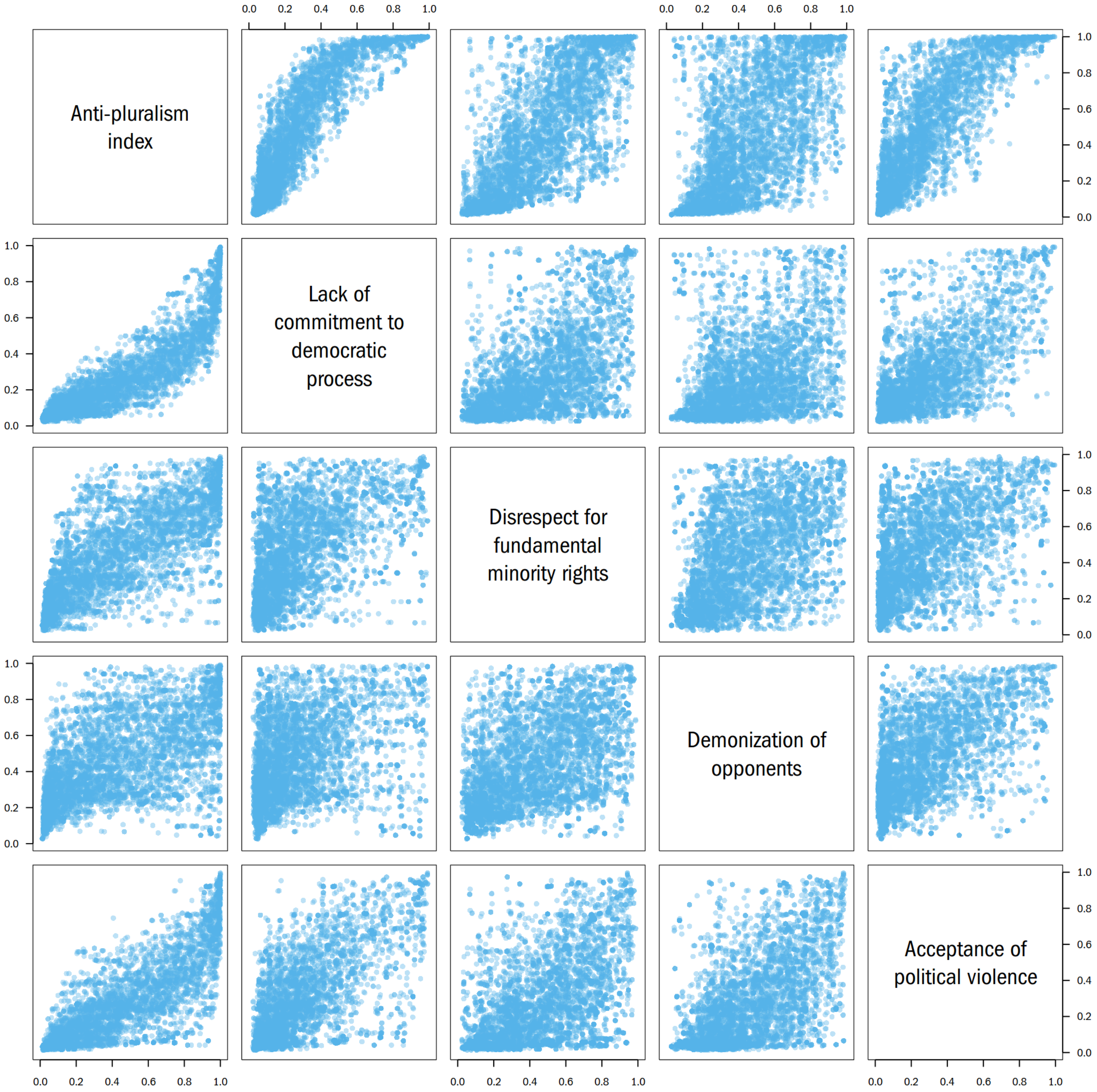

Figure A. The anti-pluralism index and its input indicators (1970-2019) in the whole V-Party dataset.

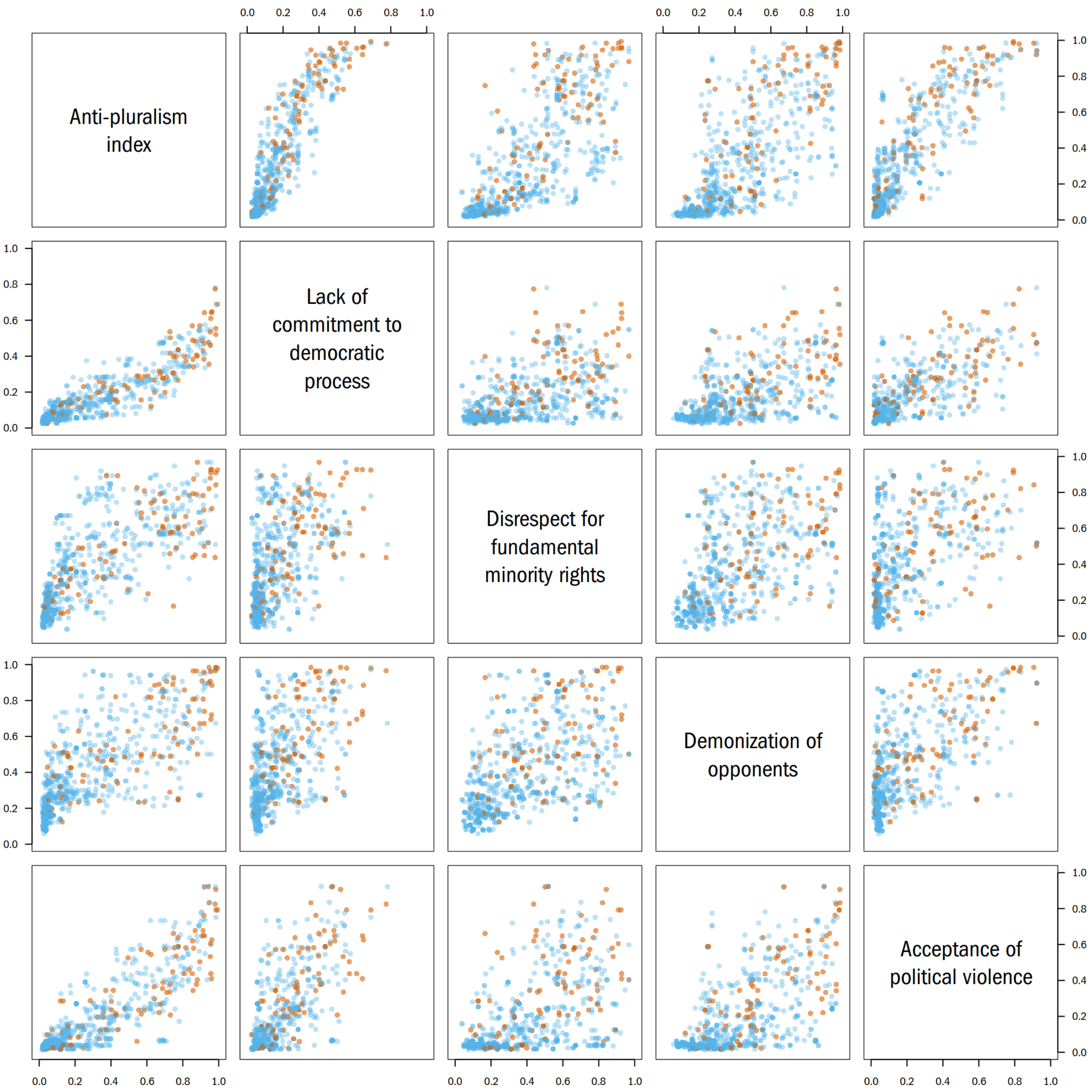

Figure B. The anti-pluralism index and its input indicators (1970-2018) in the analyzed subset of cases ($N = 771$). Observations autocratization episodes at $t + 1$ highlighted in orange.

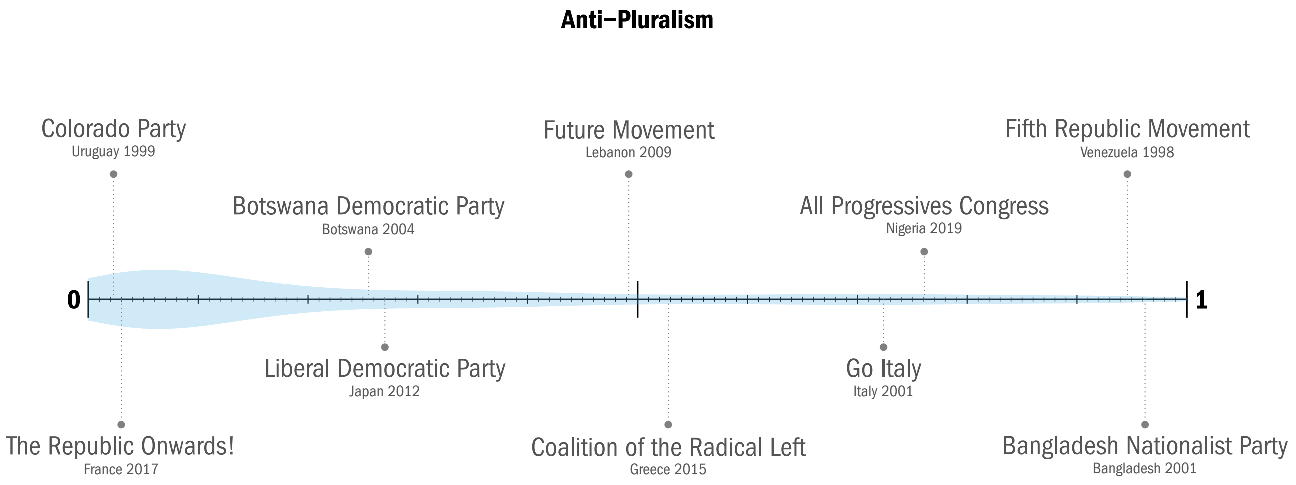

Figure C. The Anti-Pluralism Index with a density estimate in the analyzed sample ($N = 771$) and ten cases selected to illustrate different points on the scale.

Populist characteristics

Most measures of populism focus on the anti-elitism of populists. For instance, the widely used CHES data set (Polk and Rovny 2017) includes two indicators capturing the salience of anti-establishment/elite rhetoric and of anti-corruption rhetoric of political parties as well as an indicator of their position on direct democracy[24]. Norris and Inglehart (2019, p. 230) use the first two to capture their notion of populist political parties.[25] While these aspects are important for capturing populism, this choice of indicators is problematic, as it is equally important to include a measure of a notion of "the people" as homogenous and pure. As Mudde and Kaltwasser (2013, p. 150-51) note, populists do not only promote anti-elitist views, they also argue that there is a homogenous people whose general will should be realized and whom they represent. The sovereignty of the will of the homogenous and pure people over the will of the homogenous and corrupt elite is central to populist ideology, and thus solely measures of anti-elitism are not sufficient to capture populist ideologies without including the notion of group homogeneity.

In addition, the CHES indicator on direct democracy does not help much to capture populism as the cleavage direct vs. representative democracy is not at the heart of what populism is about (Müller 2017, p. 25).

The V-Party data set includes two indicators to capture populism: One that focuses on the anti-elitism rhetoric of populist parties (**Anti-Elitism**), building on Schedler (1996, p. 293) and another one on the citizen centrism of populists (**People-Centrism**). Combined to one index (**Populism**), these indicators enable the measurement of a thin version of populism.[26]

Following the arguments of Castanho Silva et al. (2020) and Wuttke et al. (2020), the V-Party data set includes a populism index that does not take the two input indexes as fully substitutable (v2xpa_popul). These recent contributions show that populism is better understood as a configuration of certain attributes rather then as their simple or weighted average, and is best measured as such. Accordingly, V-Party computes the populism score of a party in the following way. First, they rescale the OSP versions of the input variables onto the unit interval with dividing them by four. Next, the harmonic mean of these values is computed. Informally, the harmonic mean can be interpreted as in between taking the minimum as recommended by Castanho Silva et al. (2020) and taking a (weighted) arithmetic mean, but much closer to taking the minimum. In other words, this allows for some substitutability between the input indexes, but only to a very limited degree. This limited substitutability gives the resulting populism index some degree of robustness with regards to large downward errors in the input indexes. Figure D gives a density plot of the values on the Populism index.

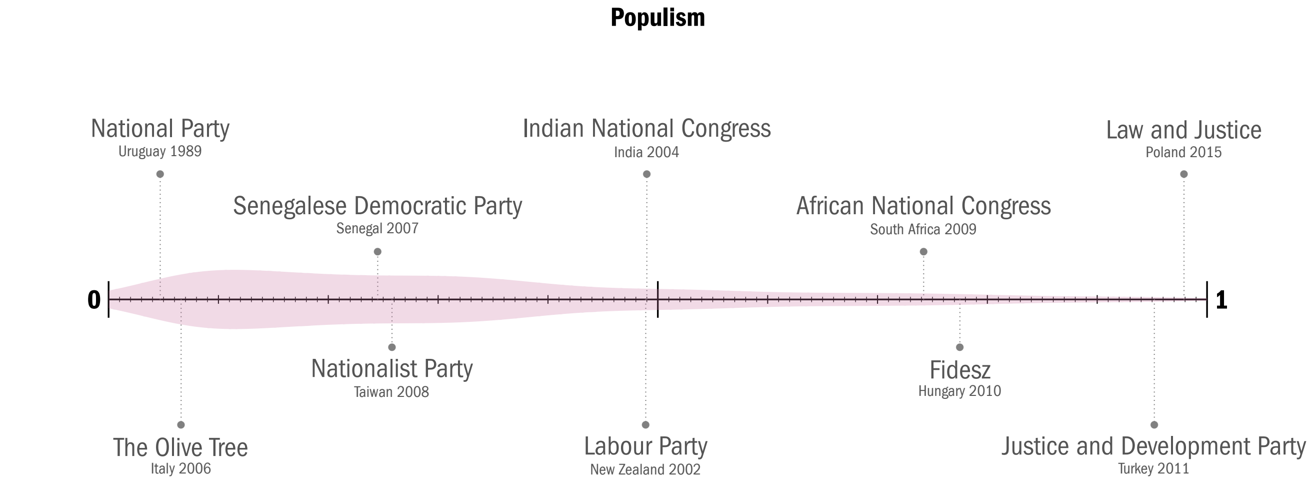

Figure D. The populism scale, with a density estimate of the scores in the analyzed sample ($N = 771$) and ten selected parties illustrating selected points on the scale.

Cultural dimension of the party system

V-Party also captures the so-called second or **cultural dimension** in the party system. This dimension has often been called GALTAN— Green, Alternative, Libertarian vs. Traditional, Authoritarian, National. These aspects have been found to correlate highly in opinion surveys (Kitschelt and Rehm 2014) and thus, expert surveys such as CHES conflate them into one question.[27]

The V-Party survey includes the following items to capture the cultural dimension:

- Immigration (v2paimmig),
- LGBT social equality (v2palgbt),
- Cultural superiority (v2paculsup),
- Religious principles (v2parelig),
- and Working women (v2pawomlab). [see Lührmann et al. (2020a) for question text]

For this paper, we aggregated them using first Bayesian Factor Analysis with a single latent factor and then used the standard normal CDF to rescale the factor scores onto the unit interval so that in the resulting index 0 stands for progressive and 1 for conservative. Figure E shows the distribution in the analyzed sample.

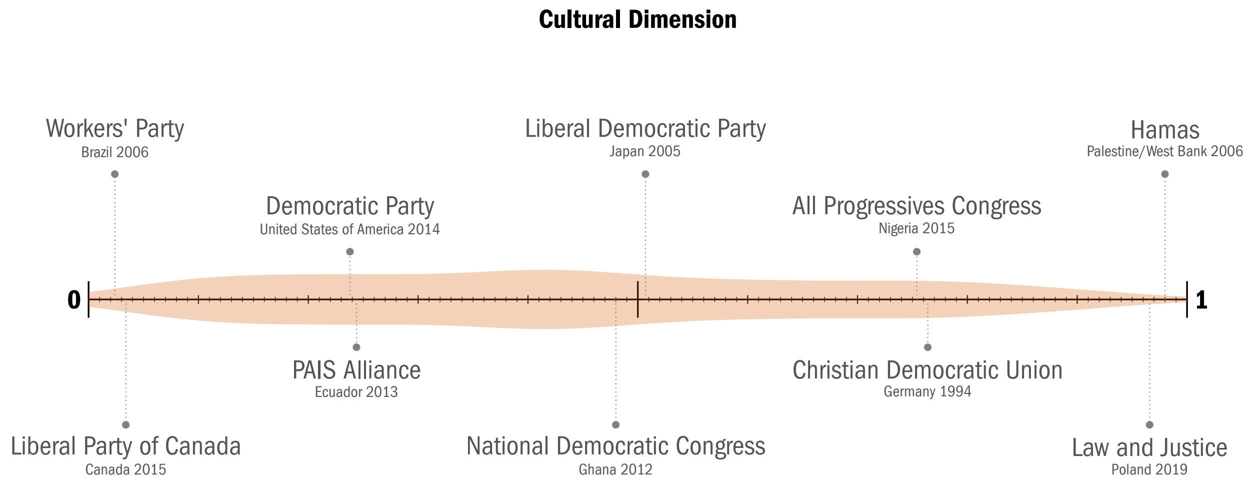

Figure E. Cultural dimension index, from least to most conservative, with a density estimate of the posterior median values in the subset of the data used in the analysis ($N = 771$). Ten selected parties highlight different points on the scale.

The economic left-right dimension in party system

Figure F gives the distribution of the data on the economic left-right dimension (v2pargilef_osp).

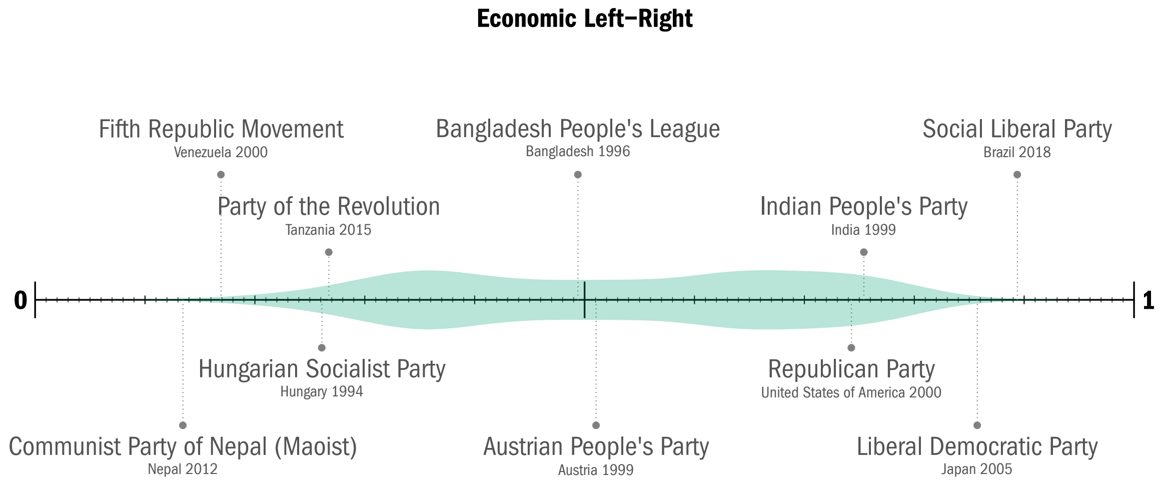

Figure F. Economic Left-Right index (v2pargilef_osp divided by 6), with a density estimate of the posterior median values in the subset of the data included in the analysis ($N = 771$). Ten selected parties highlight different points on the scale.

Multiple dimensions in the party system

Figure G gives the joint and marginal distributions of the discussed indices of party characteristics. The wide distribution of the scatter illustrates that these measures indeed capture distinct dimensions in the party system.

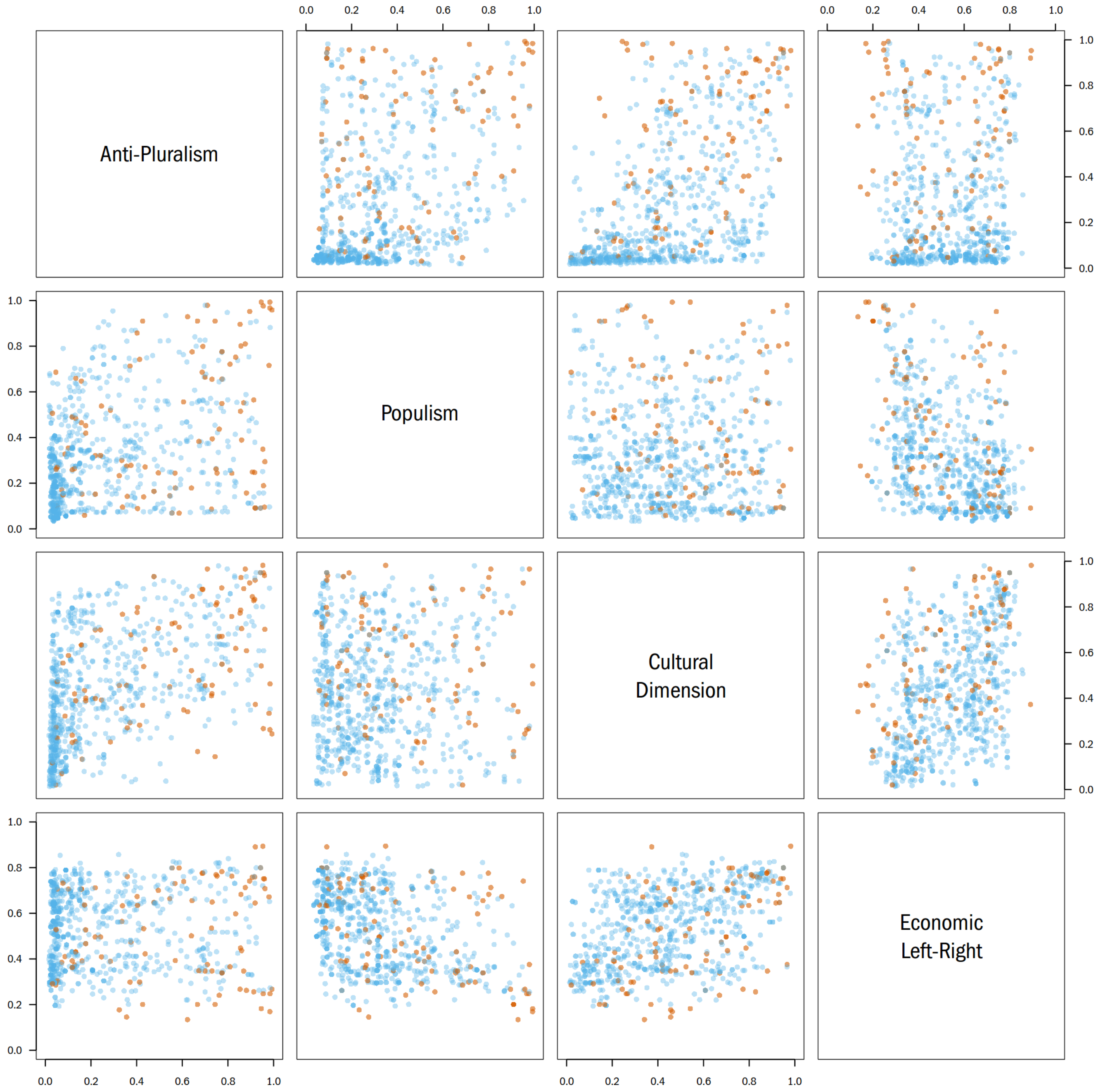

Figure G. Four indexes (1970-2019) in the analyzed subset of cases ($N = 771$). Observations autocratization episodes at $t + 1$ highlighted in orange.

The case of the United States of America 2000–19

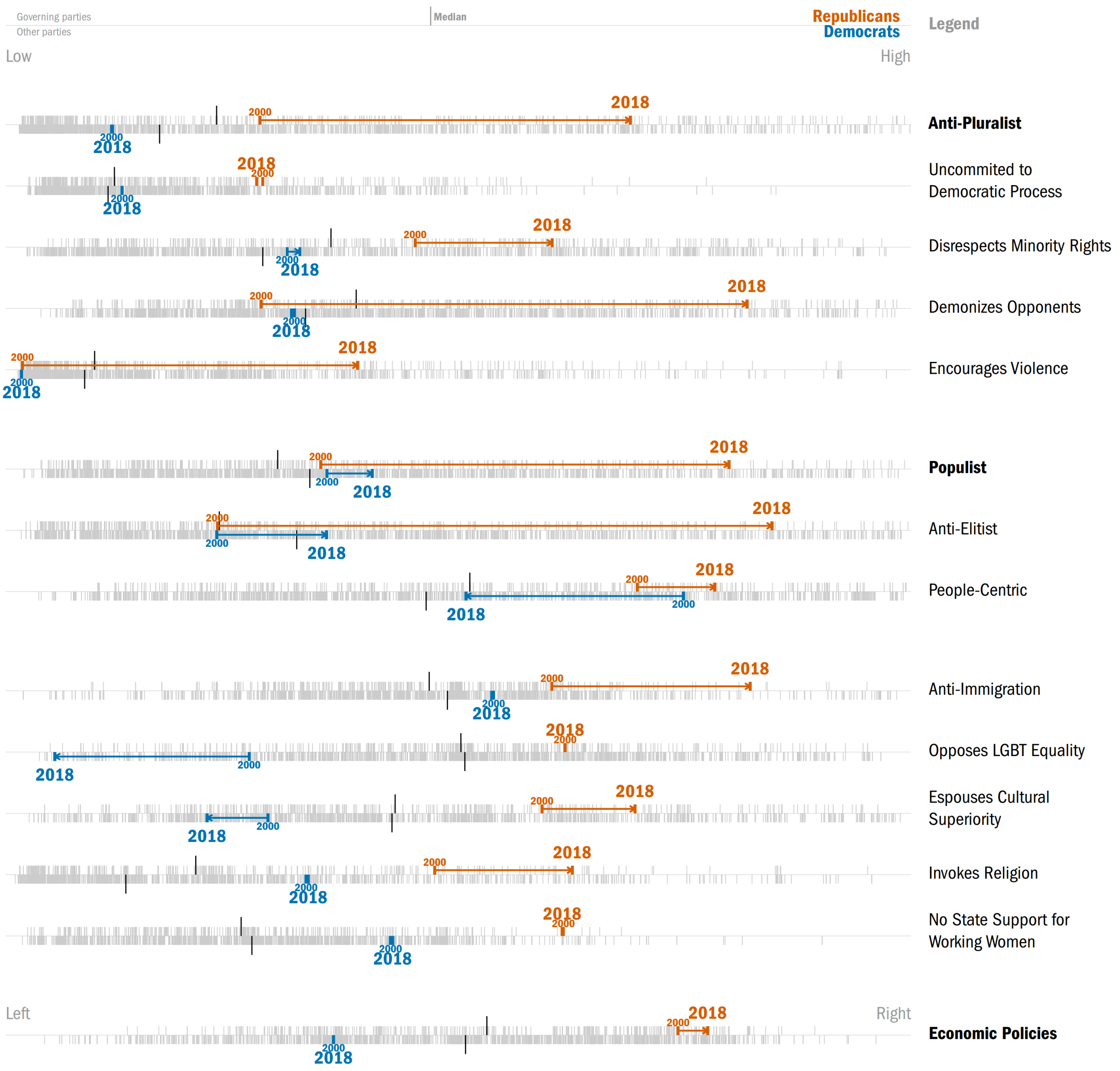

Figure H. US Democrats and Republicans 2000–18 compared on a range of indicators and indexes. Reproduced from Lührmann et al. (2020b)

Figure H illustrates that the V-Party dataset captures divergent trends over multiple dimensions in the party systems. It shows where the Republican Party (orange dots) and the Democratic Party (blue squares) placed on several indicators before the 2018 election relative to all other ruling parties in democracies (above the line) and junior governing and opposition parties in democracies (below the horizontal lines) in this millennium. The black vertical line gives the median value. The data shows that the Republican Party in 2018 was far more anti-pluralist than almost all other governing parties in democracies. Only very few governing parties in democracies in this millennium (15%) were considered more anti-pluralist than the Republican Party in

the US. Conversely, the Democratic Party was rated slightly less anti-pluralist than the typical party in democracies. The Republican party scores much higher than almost all parties in democracies on almost all of these indicators. Both parties use more populist rhetoric – anti-elitism and people-centrism – than typical parties in democracies, but the Republicans clearly more so. The five indicators capturing cultural issues place the Republicans consistently to the right of the center. Democrats also fare to the right of the median party in democracies in this millennium in terms of the role of religion in politics, immigration, and support of state measures to enhance the equal participation of women in the labor market. They fare to the left of the spectrum when it comes to support for LGBT equality and opposing the idea of cultural superiority of particular group or nation. V-Party's data places the Democratic Party to the left of typical parties in democracies in terms of economic issues and the Republican Party to the right.

Regressions

		Contextual Covariates	
		No	Yes
Component:	Lack of commitment to democratic process	.77	.83
	Disrespect for fundamental minority right	.72	.81
	Demonization of opponents	.73	.82
	Acceptance of political violence	.77	.83
Index:	Anti-Pluralism	.79	.83
	Populism	.66	.82
	Cultural Dimension	.61	.81
	Economic Left-Right	.62	.83

Table A. Predictive performance of models reported in Figure I and J. in terms of Leave-Pair-Out Area Under the Receiver Operating Characteristic Curve (LPOCV-AUC) 16 models, 4×2 single party variable with and without contextual covariates.

Dependent variable:	Autocratization in the year after election			
	(1)	(2)	(3)	(4)
Parametric coefficients				
Constant	0.79	1.05	1.02	0.77
	(0.16)	(0.15)	(0.16)	(0.17)
Liberal component ($t-1$)	−0.30	−0.51	−0.40	−0.36
	(0.13)	(0.13)	(0.13)	(0.13)
ln GDP per capita ($t-1$)	−0.05	−0.06	−0.06	−0.04
	(0.02)	(0.02)	(0.02)	(0.02)
GDP growth ($t-1$)	−0.11	−0.11	−0.14	−0.15
	(0.18)	(0.19)	(0.19)	(0.19)
Equal distribution	0.11	0.13	0.10	0.14
of resources ($t-1$)	(0.09)	(0.09)	(0.09)	(0.09)
Autocratization	0.30	0.40	0.36	0.36
episodes share ($t-1$)	(0.32)	(0.32)	(0.32)	(0.32)
Democratization	−0.46	−0.48	−0.46	−0.45
episodes share ($t-1$)	(0.19)	(0.20)	(0.19)	(0.19)
Exclusive regional EDI ($t-1$)	0.05	0.05	0.02	0.01
	(0.06)	(0.07)	(0.06)	(0.06)
Presidentialism index ($t-1$)	−0.01	−0.02	−0.01	0.00
	(0.03)	(0.03)	(0.03)	(0.03)
Smooths				
EDF: Year	1.00	1.00	1.00	1.00
	(1.00)	(1.00)	(1.00)	(1.00)
EDF: Lack of commitment to democratic process	1.00			
	(1.00)			
EDF: Disrespect for fundamental minority rights		2.74		
		(3.47)		
EDF: Demonization of opponents opponents			4.25	
			(5.27)	
EDF: Acceptance of political violence				1.00
				(1.00)
Fit				
LPOCV-AUC	0.83	0.81	0.82	0.83
AIC	316.21	338.24	331.04	325.28
BIC	371.98	402.09	401.94	381.05
Log Likelihood	−146.10	−155.38	−150.27	−150.64
Deviance	65.94	67.55	66.66	66.72
Deviance explained	0.19	0.17	0.18	0.18
Dispersion	0.09	0.09	0.09	0.09
R^2	0.18	0.15	0.16	0.17
GCV score	0.09	0.09	0.09	0.09
Num. Smooths	2	2	2	2
Num. obs.	771	771	771	771

Table B. Four Gaussian GAMs one for each component of the API, all with contextual covariates. For parametric coefficients, point estimates with standard errors in parentheses. For smooths, estimated degrees of freedom with reference degrees of freedom in parentheses. Smooths for the four components of the API shown in Figure 4.

Dependent variable:	Autocratization in the year after election			
	(5)	(6)	(7)	(8)
Parametric coefficients				
Constant	0.74	1.07	1.10	1.12
	(0.17)	(0.15)	(0.15)	(0.15)
Liberal component ($t-1$)	-0.25	-0.50	-0.50	-0.49
	(0.13)	(0.12)	(0.12)	(0.12)
ln GDP per capita ($t-1$)	-0.05	-0.06	-0.07	-0.06
	(0.02)	(0.02)	(0.02)	(0.02)
GDP growth ($t-1$)	-0.10	-0.10	-0.10	-0.08
	(0.18)	(0.18)	(0.19)	(0.18)
Equal distribution	0.11	0.14	0.18	0.12
of resources ($t-1$)	(0.09)	(0.09)	(0.09)	(0.09)
Autocratization	0.37	0.41	0.46	0.30
episodes share ($t-1$)	(0.31)	(0.32)	(0.32)	(0.32)
Democratization	-0.41	-0.46	-0.44	-0.50
episodes share ($t-1$)	(0.19)	(0.19)	(0.19)	(0.19)
Exclusive regional EDI ($t-1$)	0.01	0.01	0.03	-0.00
	(0.07)	(0.06)	(0.06)	(0.06)
Presidentialism index ($t-1$)	-0.01	-0.02	-0.01	-0.02
	(0.03)	(0.03)	(0.03)	(0.03)
Smooths				
EDF: Year	1.00	1.00	1.00	1.00
	(1.00)	(1.00)	(1.00)	(1.00)
EDF: Anti-pluralism	3.21			
	(4.03)			
EDF: Populism		8.06		
		(8.80)		
EDF: Cultural Dimension			3.28	
			(4.13)	
EDF: Economic Left-Right				7.24
				(8.31)
Fit				
LPOCV-AUC	0.83	0.82	0.81	0.83
AIC	304.97	311.91	328.31	312.39
BIC	371.02	400.47	394.67	397.17
Log Likelihood	-138.27	-136.90	-149.87	-137.96
Deviance	64.62	64.39	66.59	64.57
Deviance explained	0.20	0.21	0.18	0.20
Dispersion	0.09	0.09	0.09	0.09
R^2	0.19	0.19	0.16	0.19
GCV score	0.09	0.09	0.09	0.09
Num. obs.	771	771	771	771
Num. Smooths	2	2	2	2

Table C. Four Gaussian GAMs with contextual covariates. For parametric coefficients, point estimates with standard errors in parentheses. For smooths, estimated degrees of freedom with reference degrees of freedom in parentheses. Smooths for the four components of the API shown in Figure 5.

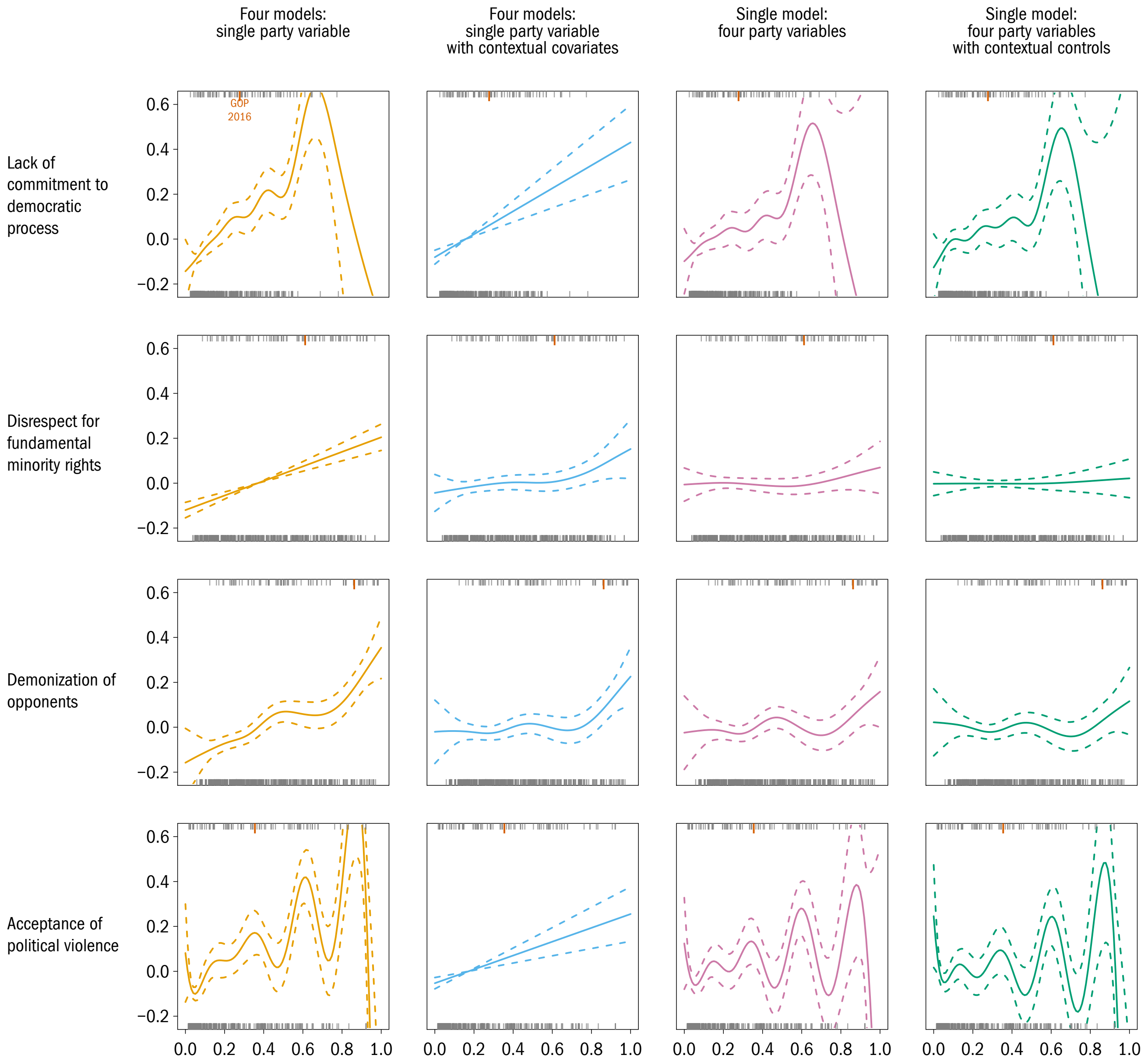

Figure I. Selected partial effects under Gaussian-identity GAMs with **autocratization episode at *t* + 1** on the left-hand-side. Dashed lines show ± 2SE regions. Upper ticks show cases with an autocratization episode, lower ticks cases without one. 2016 US Republicans highlighted with a larger orange tick. Figure rows from top to bottom: four components of anti-pluralism. Figure columns from left to right: (1) Four models with single party variable (shown), AIC: 358, 410, 388, 348. (2) Four models with single party variable (shown) and contextual covariates (not shown), AIC: 316, 338, 331, 325. (3) Single model with four party variables (shown), AIC 329. (4) Single model with four party variables (shown) and contextual covariates (not shown), AIC 299. $N = 771$, null model AIC 455, Contextual covariates only model AIC 341.

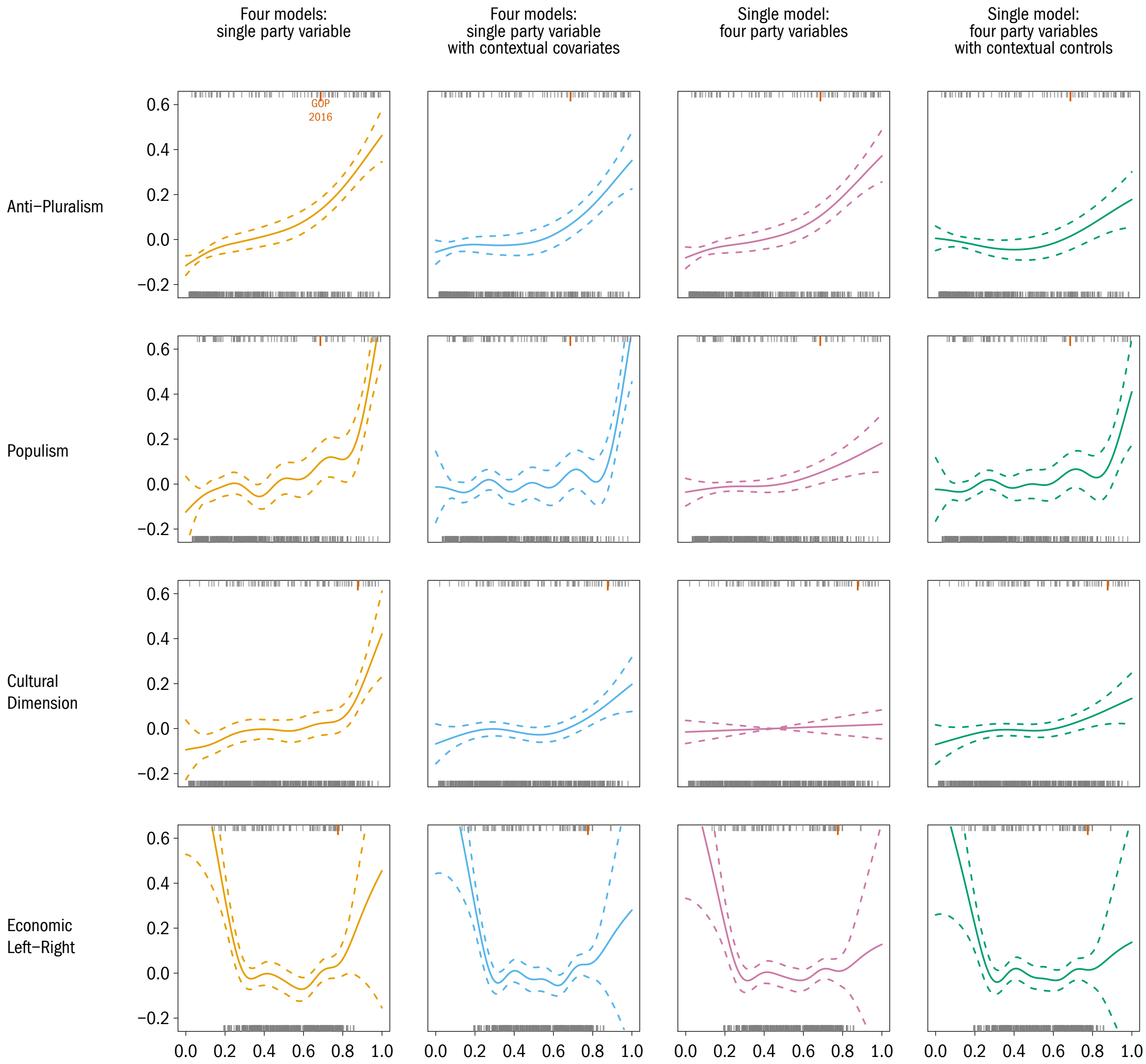

Figure J. Selected partial effects under Gaussian-identity GAMs with **autocratization episode at *t* + 1** on the left-hand-side. Dashed lines show ± 2SE regions. Upper ticks show cases with an autocratization episode, lower ticks cases without one. 2016 US Republicans highlighted with a larger orange tick. Figure rows from top to bottom: four components of anti-pluralism. Figure columns from left to right: (1) Four models with single party variable (shown), AIC: 337, 398, 429, 421. (2) Four models with single party variable (shown) and contextual covariates (not shown), AIC: 305, 312, 328, 312. (3) Single model with four party variables (shown), AIC 316. (4) Single model with four party variables (shown) and contextual covariates (not shown), AIC 276. $N = 771$, null model AIC 455, Contextual covariates only model AIC 341.

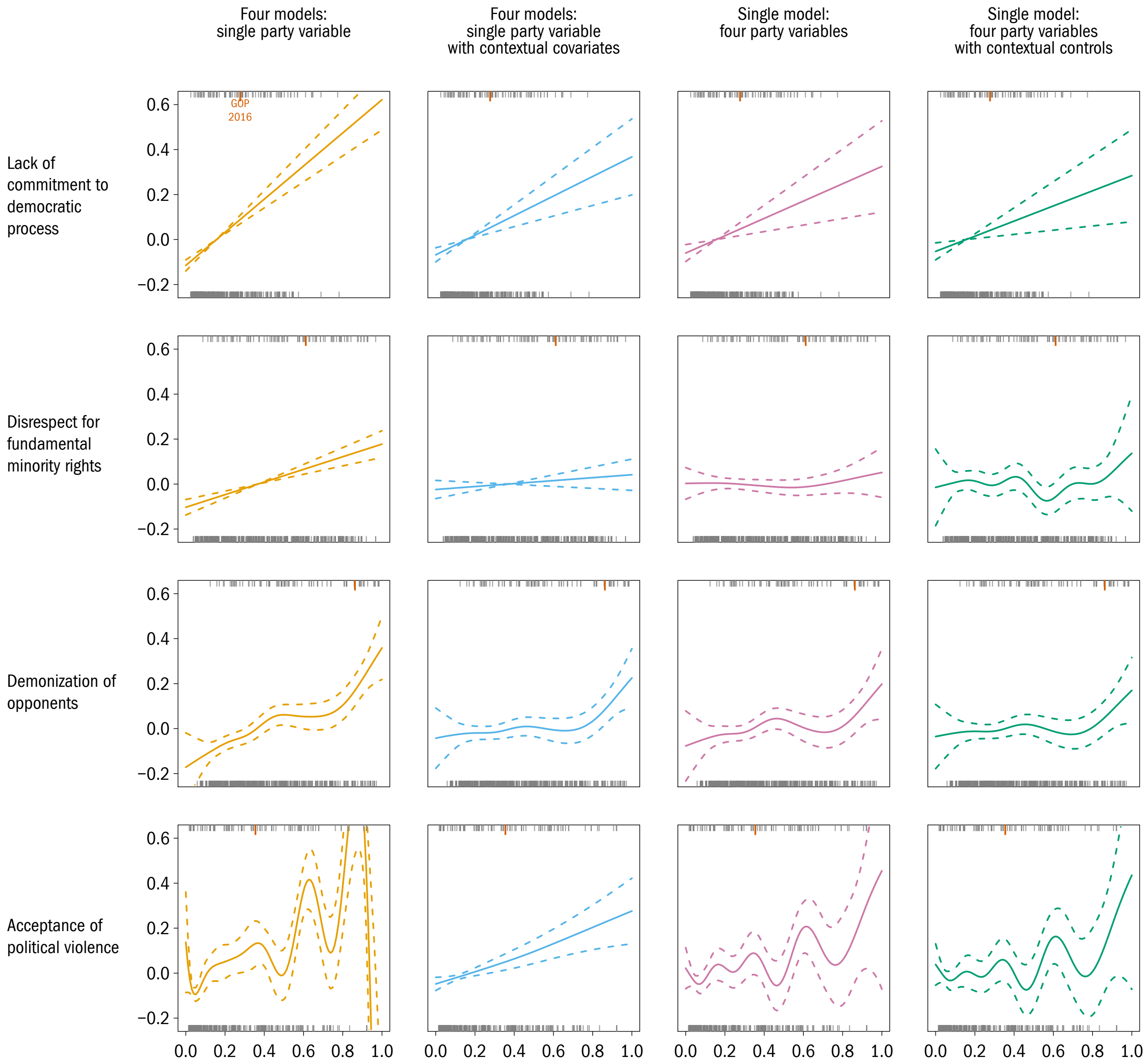

Figure K. Selected partial effects under Gaussian-identity GAMs with **autocratization episode at *t* + 2** on the left-hand-side. Dashed lines show ± 2SE regions. Upper ticks show cases with an autocratization episode, lower ticks cases without one. 2016 US Republicans highlighted with a larger orange tick. Figure rows from top to bottom: four components of anti-pluralism. Figure columns from left to right: (1) Four models with single party variable (shown), AIC: 350, 397, 369, 339. (2) Four models with single party variable (shown) and contextual covariates (not shown), AIC: 304, 321, 312, 306. (3) Single model with four party variables (shown), AIC 331. (4) Single model with four party variables (shown) and contextual covariates (not shown), AIC 299. $N = 757$.

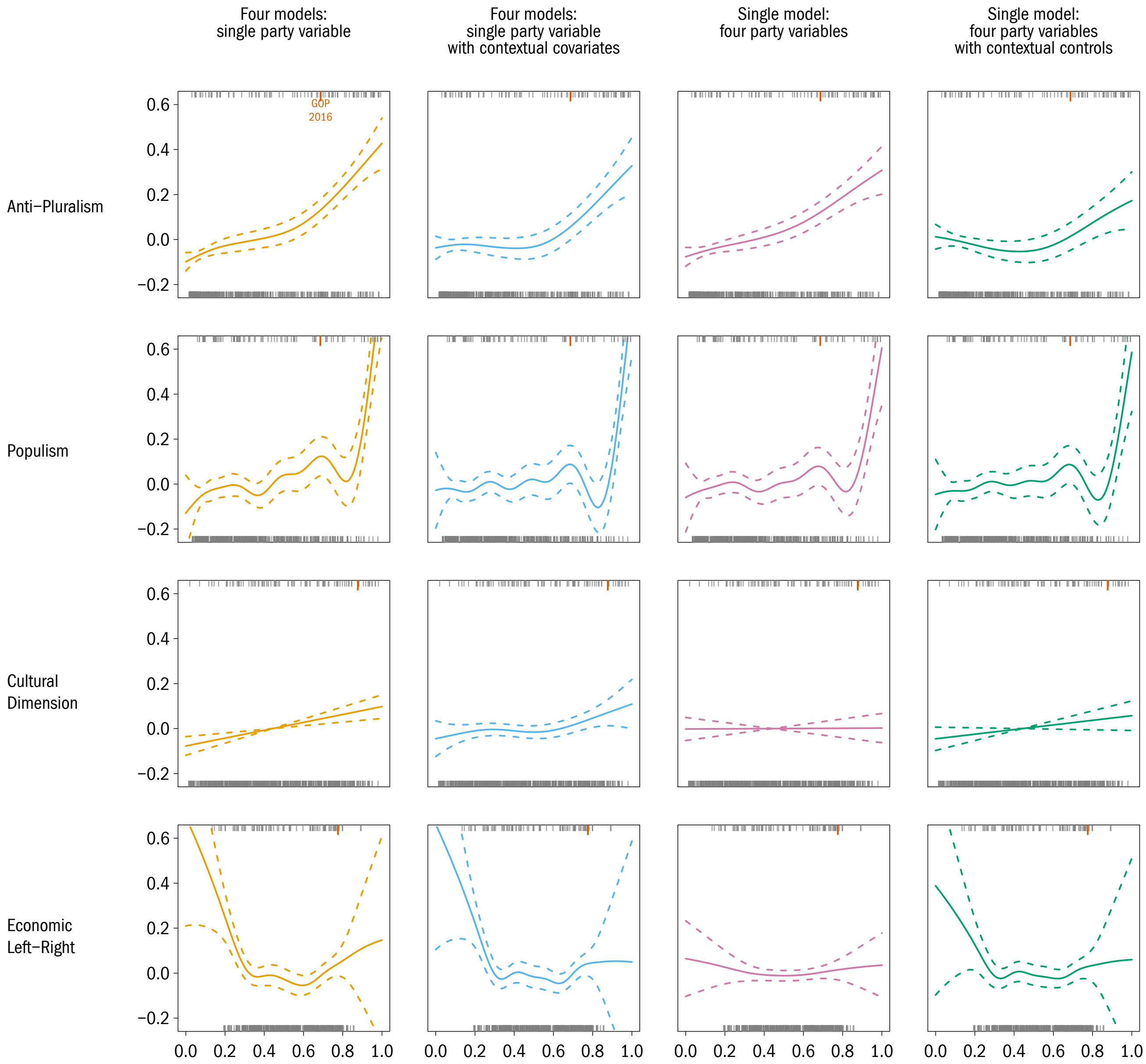

Figure L. Selected partial effects under Gaussian-identity GAMs with **autocratization episode at *t* + 2** on the left-hand-side. Dashed lines show ± 2SE regions. Upper ticks show cases with an autocratization episode, lower ticks cases without one. 2016 US Republicans highlighted with a larger orange tick. Figure rows from top to bottom: four components of anti-pluralism. Figure columns from left to right: (1) Four models with single party variable (shown), AIC: 329, 372, 418, 409. (2) Four models with single party variable (shown) and contextual covariates (not shown), AIC: 287, 286, 318, 306. (3) Single model with four party variables (shown), AIC 316. (4) Single model with four party variables (shown) and contextual covariates (not shown), AIC 267. $N = 757$.

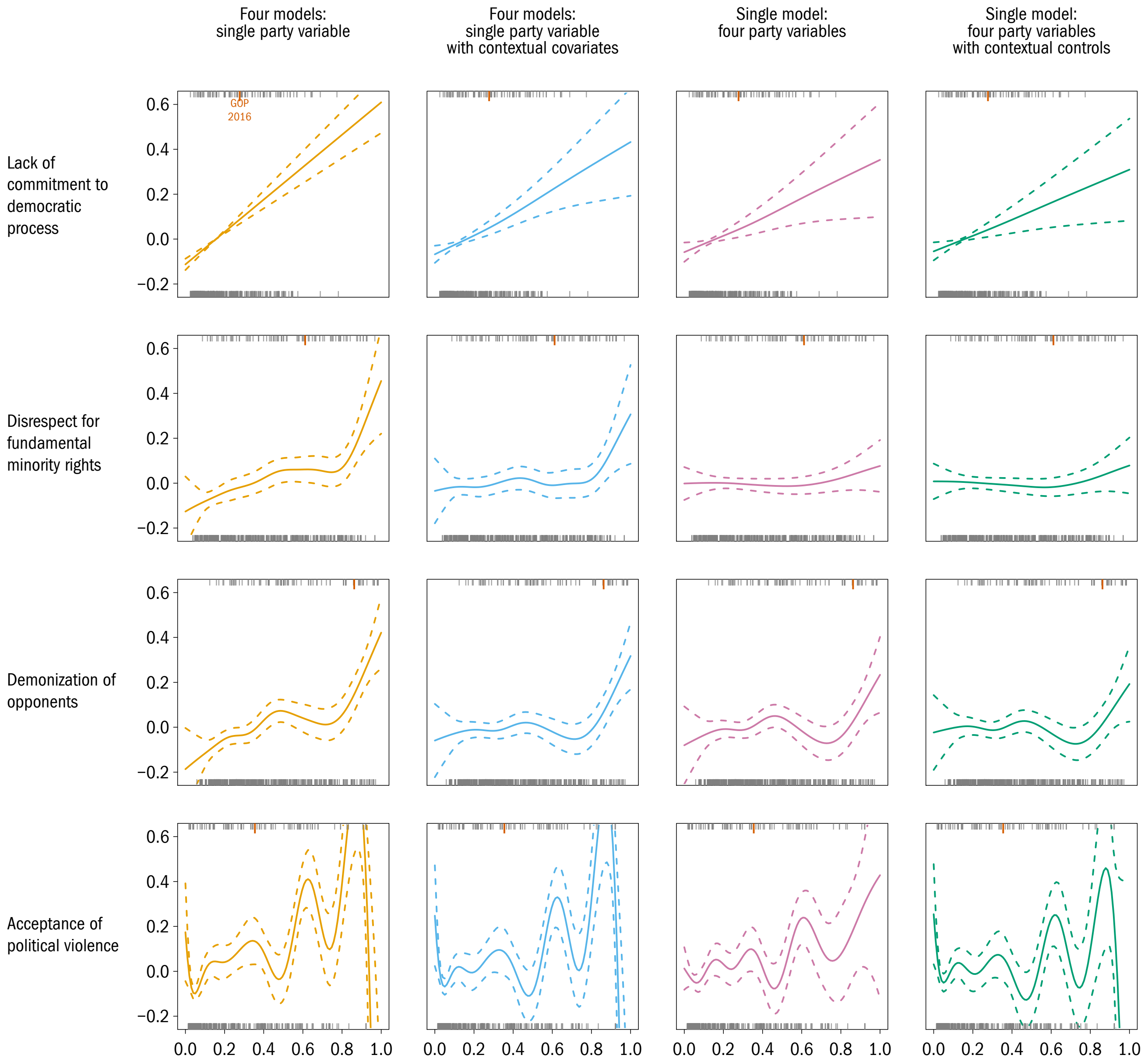

Figure M. Selected partial effects under Gaussian-identity GAMs with **autocratization episode at *t* + 3** on the left-hand-side. Dashed lines show ± 2SE regions. Upper ticks show cases with an autocratization episode, lower ticks cases without one. 2016 US Republicans highlighted with a larger orange tick. Figure rows from top to bottom: four components of anti-pluralism. Figure columns from left to right: (1) Four models with single party variable (shown), AIC: 346, 388, 363, 326. (2) Four models with single party variable (shown) and contextual covariates (not shown), AIC: 296, 314, 298, 280. (3) Single model with four party variables (shown), AIC 317. (4) Single model with four party variables (shown) and contextual covariates (not shown), AIC 272. $N = 739$.

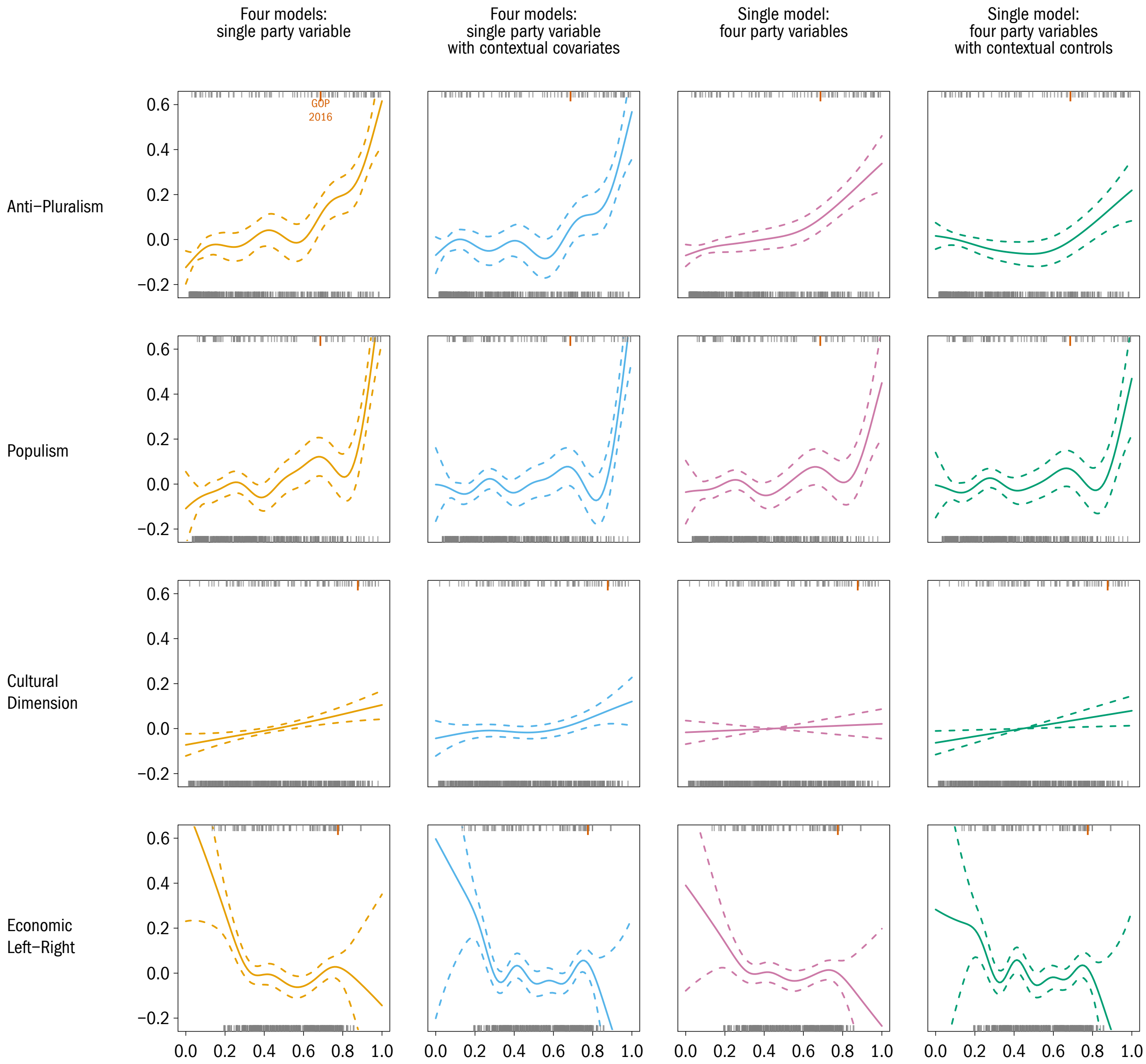

Figure N. Selected partial effects under Gaussian-identity GAMs with **autocratization episode at *t* + 3** on the left-hand-side. Dashed lines show ± 2SE regions. Upper ticks show cases with an autocratization episode, lower ticks cases without one. 2016 US Republicans highlighted with a larger orange tick. Figure rows from top to bottom: four components of anti-pluralism. Figure columns from left to right: (1) Four models with single party variable (shown), AIC: 325, 361, 409, 398. (2) Four models with single party variable (shown) and contextual covariates (not shown), AIC: 274, 278, 310, 294. (3) Single model with four party variables (shown), AIC 307. (4) Single model with four party variables (shown) and contextual covariates (not shown), AIC 248. $N = 739$.

Table D. Re-analysis of the data with Binomial-probit GLMs.

	Dependent variable: Autocratization episode at $t+1$									
	(1)	(2)	(3)	(4)	(5)	(6)	(7)	(8)	(9)	(10)
Lack of commitment to democratic process	3.36				1.99				1.73	1.46
	(0.39)				(0.54)				(0.58)	(0.64)
Disrespect for fundamental minority rights		1.59				0.57			0.38	0.06
		(0.24)				(0.32)			(0.32)	(0.36)
Demonization of opponents			1.82				0.87		0.62	0.46
			(0.24)				(0.32)		(0.33)	(0.37)
Acceptance of political violence				2.26				1.16	0.76	0.34
				(0.27)				(0.38)	(0.42)	(0.49)
Liberal Component Index ($t-1$)					−1.56	−2.59	−2.16	−2.01		−1.23
					(0.74)	(0.66)	(0.70)	(0.71)		(0.76)
ln pcGDP ($t-1$)					−0.21	−0.25	−0.29	−0.20		−0.21
					(0.11)	(0.10)	(0.10)	(0.11)		(0.11)
GDP growth ($t-1$)					−1.41	−1.56	−1.63	−1.76		−1.54
					(1.21)	(1.18)	(1.18)	(1.19)		(1.20)
Equal distribution of resources ($t-1$)					0.31	0.55	0.51	0.54		0.30
					(0.48)	(0.48)	(0.48)	(0.47)		(0.48)
Autocratization episodes share ($t-1$)					−1.64	−0.94	−1.13	−0.75		−1.28
					(4.53)	(4.45)	(4.52)	(4.50)		(4.57)
Democratization episodes share ($t-1$)					−4.09	−4.08	−3.98	−4.08		−3.79
					(2.31)	(2.26)	(2.27)	(2.30)		(2.31)
Exclusive regional EDI ($t-1$)					−0.48	−0.48	−0.47	−0.63		−0.45
					(0.46)	(0.46)	(0.46)	(0.46)		(0.48)
Presidentialism ($t-1$)					−0.04	−0.04	−0.05	0.01		−0.05
					(0.17)	(0.17)	(0.17)	(0.17)		(0.18)
Year					−1,226.93	−1,171.87	−1,201.47	−1,215.01		−1,291.76
					(469.43)	(464.08)	(469.08)	(473.64)		(478.06)
$Year^2$					0.61	0.59	0.60	0.61		0.65
					(0.24)	(0.23)	(0.23)	(0.24)		(0.24)
$Year^3$					−0.0001	−0.0001	−0.0001	−0.0001		−0.0001
					(<0.0001)	(<0.0001)	(<0.0001)	(<0.0001)		(<0.0001)
Constant	−1.84	−1.86	−2.00	−1.69	816,796.00	780,118.70	799,786.70	808,655.80	−2.13	859,824.50
	(0.10)	(0.13)	(0.13)	(0.09)	(312,527.80)	(308,984.90)	(312,315.70)	(315,345.00)	(0.16)	(318,272.40)
Observations	771	771	771	771	771	771	771	771	771	771
Log Likelihood	−243.22	−259.85	−253.04	−245.38	−207.32	−212.46	−210.35	−209.11	−235.60	−205.57
AIC	490.44	523.70	510.07	494.76	440.63	450.92	446.69	444.22	481.20	443.13

Table E. Re-analysis of the data with Binomial-probit GLMs.

	Dependent variable:									
	Autocratization episode at $t+1$									
	(1)	(2)	(3)	(4)	(5)	(6)	(7)	(8)	(9)	(10)
Anti-pluralism	1.90				1.17				1.67	0.67
	(0.21)				(0.31)				(0.26)	(0.37)
Populism		1.36				0.84			0.67	0.72
		(0.25)				(0.28)			(0.30)	(0.33)
Cultural dimension			1.11				0.82		0.19	0.63
			(0.24)				(0.30)		(0.35)	(0.39)
Economic left-right				−0.33				−0.14	−0.20	−0.09
				(0.34)				(0.40)	(0.45)	(0.53)
Liberal Component Index ($t-1$)					−1.52	−2.87	−2.56	−2.93		−1.82
					(0.74)	(0.65)	(0.65)	(0.64)		(0.75)
ln GDPpc ($t-1$)					−0.19	−0.30	−0.29	−0.27		−0.24
					(0.11)	(0.10)	(0.10)	(0.11)		(0.12)
GDP growth ($t-1$)					−1.65	−1.69	−1.64	−1.56		−1.85
					(1.19)	(1.17)	(1.18)	(1.19)		(1.18)
Equal distribution of resources ($t-1$)					0.38	0.70	0.64	0.62		0.54
					(0.48)	(0.48)	(0.48)	(0.48)		(0.49)
Autocratization episodes share ($t-1$)					−0.77	−0.81	−0.97	−1.33		−0.38
					(4.52)	(4.50)	(4.49)	(4.45)		(4.56)
Democratization episodes share ($t-1$)					−3.82	−4.23	−4.41	−4.41		−3.96
					(2.29)	(2.25)	(2.29)	(2.27)		(2.28)
Exclusive regional EDI ($t-1$)					−0.47	−0.36	−0.44	−0.61		−0.22
					(0.46)	(0.46)	(0.45)	(0.45)		(0.48)
Presidentialism ($t-1$)					−0.05	−0.06	−0.005	−0.01		−0.07
					(0.17)	(0.17)	(0.17)	(0.17)		(0.17)
Year					−1,285.19	−1,055.57	−1,249.20	−1,091.19		−1,254.73
					(476.96)	(461.06)	(466.31)	(462.26)		(477.57)
Year^2					0.64	0.53	0.63	0.55		0.63
					(0.24)	(0.23)	(0.23)	(0.23)		(0.24)
Year^3					−0.0001	−0.0001	−0.0001	−0.0001		−0.0001
					(<0.0001)	(<0.0001)	(<0.0001)	(<0.0001)		(<0.0001)
Constant	−1.87	−1.67	−1.71	−1.01	855,319.00	702,703.00	831,566.80	726,514.20	−2.02	835,004.90
	(0.11)	(0.11)	(0.14)	(0.19)	(317,543.40)	(306,974.00)	(310,457.90)	(307,783.40)	(0.28)	(317,947.30)
Observations	771	771	771	771	771	771	771	771	771	771
Log Likelihood	−236.92	−265.94	−271.01	−281.38	−206.69	−209.41	−210.18	−214.02	−233.42	−202.82
AIC	477.84	535.89	546.02	566.75	439.38	444.81	446.36	454.04	476.83	437.64

Table F. OLS regressions. Left-hand-side: Revised Polity score (`e_polity2`) at last pre-election year ($t-1$) and first post-election year ($t+1$).

	Dependent variable:									
	Change in `e_polity2` from $t-1$ to $t+1$									
	(1)	(2)	(3)	(4)	(5)	(6)	(7)	(8)	(9)	(10)
Lack of commitment to democratic process	−0.62 (0.35)				−1.09 (0.45)				−0.72 (0.53)	−0.82 (0.54)
Disrespect for fundamental minority rights		0.02 (0.19)				0.01 (0.24)			0.25 (0.24)	0.22 (0.26)
Demonization of opponents			0.005 (0.21)				−0.02 (0.26)		0.40 (0.29)	0.38 (0.30)
Acceptance of political violence				−0.44 (0.25)				−0.95 (0.35)	−0.54 (0.39)	−0.93 (0.43)
Liberal Component Index ($t-1$)					0.10 (0.60)	0.61 (0.58)	0.59 (0.60)	0.13 (0.59)		0.18 (0.62)
ln pcGDP ($t-1$)					−0.13 (0.08)	−0.10 (0.08)	−0.10 (0.08)	−0.16 (0.09)		−0.17 (0.09)
GDP growth ($t-1$)					0.11 (0.84)	0.08 (0.85)	0.09 (0.85)	0.24 (0.84)		0.15 (0.84)
Equal distribution of resources ($t-1$)					0.07 (0.38)	−0.002 (0.39)	0.001 (0.39)	−0.03 (0.38)		−0.03 (0.39)
Autocratization episodes share ($t-1$)					4.56 (3.04)	4.51 (3.05)	4.51 (3.05)	4.39 (3.04)		4.61 (3.04)
Democratization episodes share ($t-1$)					−1.21 (0.99)	−1.16 (0.99)	−1.17 (0.99)	−1.24 (0.98)		−1.22 (0.98)
Exclusive regional EDI ($t-1$)					0.05 (0.27)	0.14 (0.29)	0.13 (0.28)	0.12 (0.27)		0.19 (0.29)
Presidentialism ($t-1$)					0.12 (0.13)	0.10 (0.13)	0.10 (0.13)	0.09 (0.13)		0.08 (0.13)
Year					300.10 (307.18)	298.61 (308.49)	299.03 (308.49)	294.43 (306.83)		287.82 (306.61)
Year^2					−0.15 (0.15)	−0.15 (0.15)	−0.15 (0.15)	−0.15 (0.15)		−0.14 (0.15)
Year^3					$0<\beta<0.0001$ (<0.0001)	$0<\beta<0.0001$ (<0.0001)	$0<\beta<0.0001$ (<0.0001)	$0<\beta<0.0001$ (<0.0001)		$0<\beta<0.0001$ (<0.0001)
Constant	0.13 (0.07)	0.03 (0.08)	0.04 (0.09)	0.11 (0.06)	−201,288.10 (204,548.70)	−200,363.00 (205,420.30)	−200,635.50 (205,420.30)	−197,478.20 (204,316.20)	−0.01 (0.10)	−193,142.70 (204,170.90)
Observations	698	698	698	698	698	698	698	698	698	698
Log Likelihood	−1,140.23	−1,141.79	−1,141.80	−1,140.30	−1,135.93	−1,138.87	−1,138.86	−1,135.13	−1,137.99	−1,133.00
AIC	2,284.46	2,287.59	2,287.60	2,284.61	2,297.87	2,303.73	2,303.73	2,296.26	2,285.98	2,298.00

Table G. OLS regressions. Left-hand-side: difference in Revised Polity score (`e_polity2`) at last pre-election year ($t-1$) and first post-election year ($t+1$).

	Dependent variable:									
	Change in `e_polity2` from $t-1$ to $t+1$									
	(1)	(2)	(3)	(4)	(5)	(6)	(7)	(8)	(9)	(10)
Anti-pluralism	−0.20				−0.48				−0.58	−0.89
	(0.17)				(0.25)				(0.22)	(0.29)
Populism		−0.25				−0.34			−0.39	−0.49
		(0.21)				(0.23)			(0.25)	(0.26)
Cultural dimension			0.40				0.46		1.15	1.27
			(0.19)				(0.21)		(0.25)	(0.26)
Economic left-right				−0.46				−0.46	−1.43	−1.57
				(0.28)				(0.29)	(0.35)	(0.37)
Liberal Component Index ($t-1$)					0.16	0.58	0.77	0.55		0.07
					(0.61)	(0.56)	(0.57)	(0.57)		(0.60)
ln pcGDP ($t-1$)					−0.13	−0.11	−0.10	−0.07		−0.10
					(0.08)	(0.08)	(0.08)	(0.08)		(0.09)
GDP growth ($t-1$)					0.19	0.13	0.02	0.06		0.08
					(0.84)	(0.84)	(0.84)	(0.84)		(0.83)
Equal distribution of resources ($t-1$)					0.05	−0.01	0.06	−0.03		0.14
					(0.39)	(0.38)	(0.39)	(0.39)		(0.38)
Autocratization episodes share ($t-1$)					4.40	4.44	4.60	4.55		4.54
					(3.05)	(3.05)	(3.04)	(3.05)		(2.99)
Democratization episodes share ($t-1$)					−1.21	−1.19	−1.10	−1.19		−1.21
					(0.99)	(0.99)	(0.99)	(0.99)		(0.97)
Exclusive regional EDI ($t-1$)					0.04	0.08	0.23	0.09		−0.04
					(0.28)	(0.28)	(0.27)	(0.27)		(0.28)
Presidentialism ($t-1$)					0.12	0.12	0.09	0.11		0.15
					(0.13)	(0.13)	(0.13)	(0.13)		(0.13)
Year					303.31	273.82	260.77	333.85		286.47
					(307.68)	(308.42)	(307.86)	(308.70)		(302.87)
Year^2					−0.15	−0.14	−0.13	−0.17		−0.14
					(0.15)	(0.15)	(0.15)	(0.15)		(0.15)
Year^3					$0< \beta <0.0001$	$0< \beta <0.0001$	$0< \beta <0.0001$	$0< \beta <0.0001$		$0< \beta <0.0001$
					(<0.0001)	(<0.0001)	(<0.0001)	(<0.0001)		(<0.0001)
Constant	0.09	0.12	−0.13	0.28	−203,378.20	−183,850.60	−175,190.30	−223,892.80	0.57	−192,387.50
	(0.07)	(0.08)	(0.10)	(0.16)	(204,879.40)	(205,376.30)	(204,998.30)	(205,563.20)	(0.20)	(201,683.30)
Observations	698	698	698	698	698	698	698	698	698	698
Log Likelihood	−1,141.13	−1,141.07	−1,139.68	−1,140.43	−1,137.04	−1,137.73	−1,136.38	−1,137.59	−1,128.12	−1,122.29
AIC	2,286.27	2,286.15	2,283.35	2,284.87	2,300.09	2,301.46	2,298.76	2,301.17	2,266.24	2,276.59

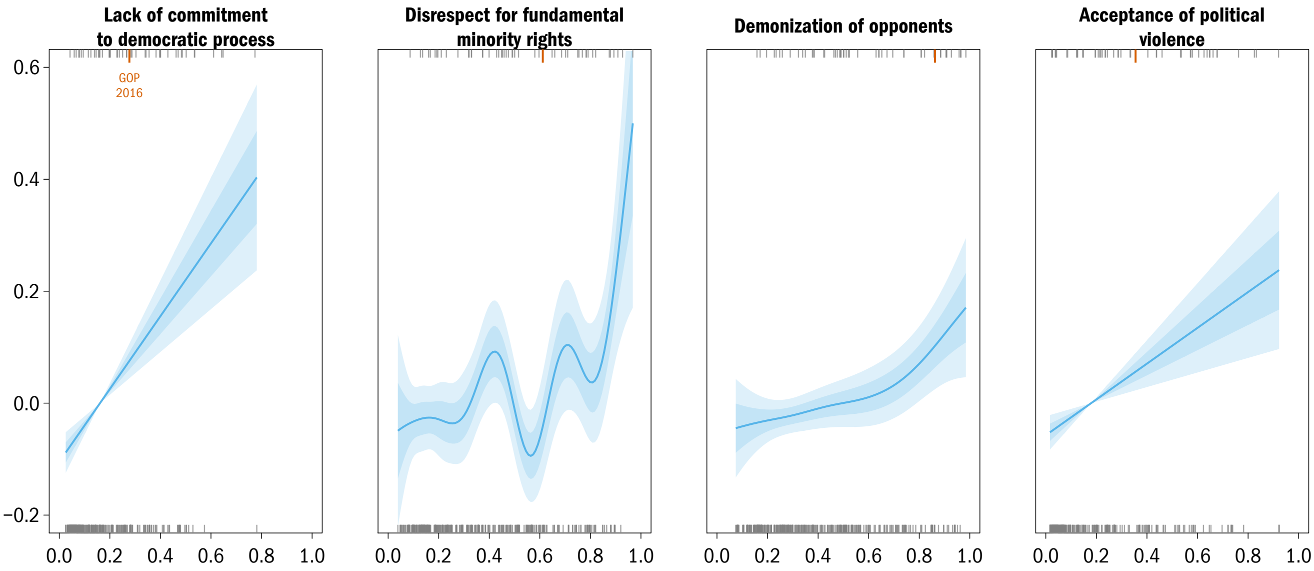

Figure O. Modeled Probability of Autocratization in the Year after Election (Indicator-level). Partial effects under Gaussian identity-link GAMs with GP smooths. Only new incumbents included ($N = 421$). Each model includes the plotted index and adjusts for the same set of contextual coviariates (see above). Upper rugs show observations with autocratization at $t+1$, lower rugs observations without it. 2016 US Republicans highlighted with a longer orange tick. Shaded ± 1SE and ± 2SE regions. Model summaries reported in Table H.

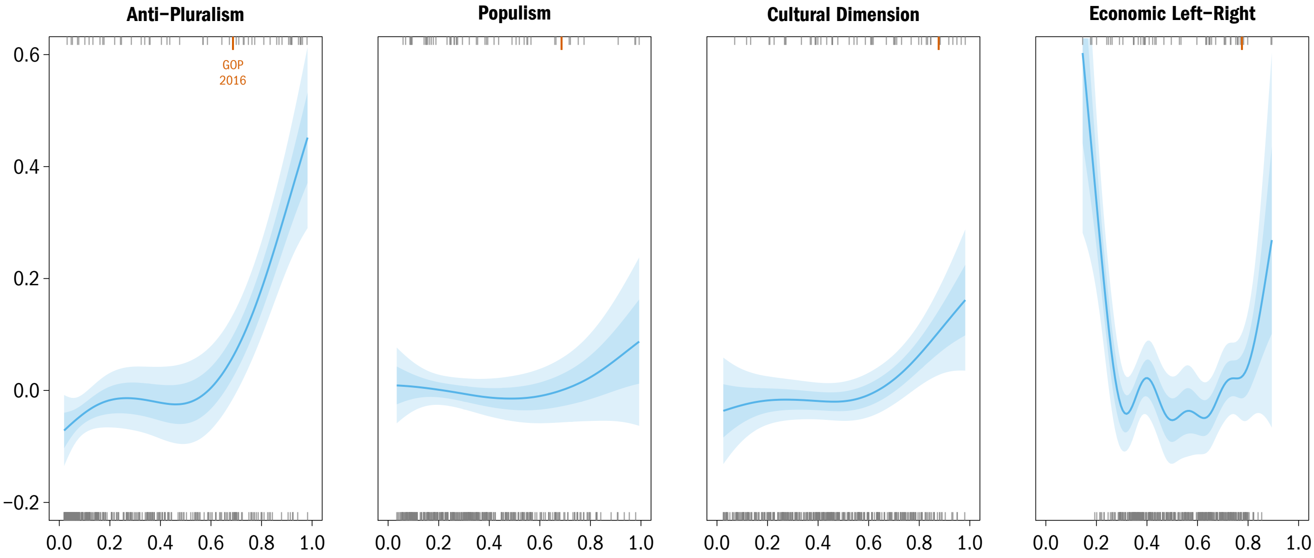

Figure P. Modeled Probability of Autocratization in the Year after Election (Index-level) Partial effects under four Gaussian identity-link GAMs. Only new incumbents included ($N = 421$). Each model includes the plotted index and adjusts for the same set of covariates. Upper rugs show observations with autocratization at $t+1$, lower rugs observations without it. 2016 US Republicans highlighted with a longer orange tick. Shaded ± 1SE and ± 2SE regions. Model summaries reported in Table I.

Dependent variable:	Autocratization in the year after election			
	(1)	(2)	(3)	(4)
Parametric coefficients				
Constant	0.86	1.17	1.10	0.91
	(0.22)	(0.22)	(0.23)	(0.24)
Liberal component $(t-1)$	-0.09	-0.37	-0.32	-0.24
	(0.18)	(0.18)	(0.18)	(0.18)
ln GDP per capita $(t-1)$	(0.03)	(0.03)	(0.03)	(0.03)
GDP growth $(t-1)$	-0.31	-0.33	-0.36	-0.42
	(0.24)	(0.25)	(0.25)	(0.25)
Equal distribution of resources $(t-1)$	0.12	0.14	0.16	0.17
	(0.12)	(0.13)	(0.12)	(0.12)
Autocratization episodes share $(t-1)$	0.28	0.38	0.42	0.34
	(0.44)	(0.44)	(0.45)	(0.44)
Democratization episodes share $(t-1)$	-0.45	-0.52	-0.45	-0.46
	(0.27)	(0.27)	(0.27)	(0.27)
Exclusive regional EDI $(t-1)$	0.14	0.11	0.10	0.08
	(0.08)	(0.09)	(0.09)	(0.08)
Presidentialism index $(t-1)$	0.00	-0.03	0.01	0.02
	(0.04)	(0.04)	(0.04)	(0.04)
Smooths				
EDF: Year	1.00	1.00	1.00	1.00
	(1.00)	(1.00)	(1.00)	(1.00)
EDF: Lack of commitment to democratic process	1.00			
	(1.00)			
EDF: Disrespect for fundamental minority rights		7.71		
		(8.61)		
EDF: Demonization of opponents			2.40	
			(3.06)	
EDF: Acceptance of political violence				1.00
				(1.00)
Fit				
AIC	199.16	212.35	216.07	211.21
BIC	247.67	288.00	270.23	259.72
Log Likelihood	-87.58	-87.46	-94.63	-93.61
Deviance	37.37	37.35	38.64	38.45
Deviance explained	0.18	0.18	0.15	0.16
Dispersion	0.09	0.09	0.09	0.09
R^2	0.16	0.15	0.13	0.14
GCV score	0.09	0.10	0.10	0.10
Num. obs.	421	421	421	421
Num. Smooths	2	2	2	2

Table H. Four Gaussian GAMs one for each component of the API, all with contextual covariates. For parametric coefficients, point estimates with standard errors in parentheses. For smooths, estimated degrees of freedom with reference degrees of freedom in parentheses. Smooths for the four components of the API shown in Figure O.

Dependent variable:	Autocratization in the year after election			
	(5)	(6)	(7)	(8)
Parametric coefficients				
Constant	0.79	1.27	1.21	1.28
	(0.24)	(0.22)	(0.22)	(0.21)
Liberal component ($t-1$)	−0.05	−0.44	−0.37	−0.42
	(0.18)	(0.17)	(0.17)	(0.17)
ln GDP per capita ($t-1$)	−0.08	−0.10	−0.10	−0.10
	(0.03)	(0.03)	(0.03)	(0.03)
GDP growth ($t-1$)	−0.25	−0.32	−0.35	−0.22
	(0.24)	(0.25)	(0.25)	(0.25)
Equal distribution of resources	0.15	0.20	0.19	0.19
	(0.12)	(0.12)	(0.12)	(0.12)
Autocratization episodes share ($t-1$)	0.35	0.37	0.35	0.26
	(0.43)	(0.45)	(0.45)	(0.44)
Democratization episodes share ($t-1$)	−0.39	−0.52	−0.49	−0.54
	(0.26)	(0.28)	(0.27)	(0.27)
Exclusive regional EDI ($t-1$)	0.09	0.05	0.07	0.06
	(0.09)	(0.09)	(0.09)	(0.08)
Presidentialism index ($t-1$)	0.01	0.01	0.01	0.00
	(0.04)	(0.04)	(0.04)	(0.04)
Smooths				
EDF: Year	1.00	1.00	1.00	1.00
	(1.00)	(1.00)	(1.00)	(1.00)
EDF: Anti-pluralism	3.34			
	(4.17)			
EDF: Populism		2.09		
		(2.68)		
EDF: Cultural dimension			2.43	
			(3.11)	
EDF: Economic Left-Right				7.10
				(8.20)
Fit				
AIC	188.55	222.11	216.35	206.48
BIC	246.50	275.04	270.65	279.66
Log Likelihood	−79.94	−97.96	−94.74	−85.14
Deviance	36.04	39.26	38.66	36.94
Deviance explained	0.21	0.14	0.15	0.19
Dispersion	0.09	0.10	0.09	0.09
R^2	0.19	0.12	0.13	0.16
GCV score	0.09	0.10	0.10	0.10
Num. obs.	421	421	421	421
Num. Smooths	2	2	2	2

Table I. Four Gaussian GAMs with contextual covariates. For parametric coefficients, point estimates with standard errors in parentheses. For smooths, estimated degrees of freedom with reference degrees of freedom in parentheses. Smooths for the four components of the API shown in Figure O.

References

Adcock R and Collier D (2001) Measurement validity: A shared standard for qualitative and quantitative research. *American Political Science Review* 95(3): 529–546.

Airola A, Pahikkala T, Waegeman W, De Baets B and Salakoski T (2009) A comparison of AUC estimators in small-sample studies. In: *Machine Learning in Systems Biology*. PMLR, pp. 3–13.

Akkerman T (2003) Populism and democracy: Challenge or pathology? *Acta Politica* 38(2): 147–159.

Akkerman T and Rooduijn M (2015) Pariahs or partners? Inclusion and exclusion of radical right parties and the effects on their policy positions. *Political Studies* 63(5): 1140–1157.

Alarcon B, Alvarez AE and Hidalgo M (2016) Latin America's new turbulence: Can democracy win in Venezuela? *Journal of Democracy* 27(2): 20–34.

Aldrich JH (2011) *Why parties? A second look*. University of Chicago Press.

Angrist JD and Pischke JS (2008) *Mostly harmless econometrics: An empiricist's companion*. Princeton University Press.

Aslanidis P and Rovira Kaltwasser C (2016) Dealing with populists in government: the SYRIZA-ANEL coalition in Greece. *Democratization* 23(6): 1077–1091.

Bakker R, De Vries C, Edwards E, Hooghe L, Jolly S, Marks G, Polk J, Rovny J, Steenbergen M and Vachudova MA (2015) Measuring party positions in Europe: The Chapel Hill expert survey trend file, 1999–2010. *Party Politics* 21(1): 143–152.

Bermeo N (2003) *Ordinary people in extraordinary times: The citizenry and the breakdown of democracy*. Princeton University Press.

Bermeo N (2016) On democratic backsliding. *Journal of Democracy* 27(1): 5–19.

Bernhard M, Nordstrom T and Reenock C (2001) Economic performance, institutional intermediation, and democratic survival. *Journal of Politics* 63(3): 775–803.

Boese VA, Edgell AB, Hellmeier S, Maerz SF and Lindberg SI (2021) How democracies prevail: Democratic resilience as a two-stage process0. *Democratization* 28(5): 885–907.

Bolt J, Inklaar R, de Jong H and Luiten van Zanden J (2018) Maddison project database, version 2018. *Maddison Project Working paper* 10.

Cassani A and Tomini L (2020) Reversing regimes and concepts: from democratization to autocratization. *European Political Science* 19(2): 272–287.

Castanho Silva B, Jungkunz S, Helbling M and Littvay L (2020) An empirical comparison of seven populist attitudes scales. *Political Research Quarterly* 73(2): 409–424.

Chalhoub S, Collins C, Llanos M, Pachón M and Perry KKY (2017) Report of the LASA fact-finding delegation on the impeachment of Brazilian president Dilma Rousseff. *Latin American Studies Association* .

Coppedge M, Gerring J, Knutsen CH, Lindberg SI, Teorell J, Altman D, Bernhard M, Fish MS, Glynn A, Hicken A et al. (2020) V-Dem Codebook v10 .

Corrales J (2020) Authoritarian survival: Why Maduro hasn't fallen. *Journal of Democracy* 31(3): 39–53.

Dahl R (1971) *Polyarchy: Participation and opposition*. Yale University Press.

Diamond L (2020) Democratic regression in comparative perspective: scope, methods, and causes. *Democratization* 28(1): 1–21.

Galston WA (2017) *Anti-pluralism: The populist threat to liberal democracy*. Yale University Press.

Galston WA (2018) The populist challenge to liberal democracy. *Journal of Democracy* 29(2): 5–19.

Ganev VI (2018) Explaining Eastern Europe: "soft decisionism" in Bulgaria. *Journal of Democracy* 29(3): 91–103.

Ganguly S (2020) An illiberal India? *Journal of Democracy* 31(1): 193–202.

Graham MH and Svolik MW (2020) Democracy in america? partisanship, polarization, and the robustness of support for democracy in the united states. *American Political Science Review* 114(2): 392409.

Haggard S and Kaufman RR (2016) *Dictators and democrats: Masses, elites, and regime change*. Princeton University Press.

Harper J (2010) Negating negation: Civic Platform, Law and Justice, and the struggle over "Polishness". *Problems of Post-Communism* 57(4): 16–36.

Hastie TJ and Tibshirani RJ (1990) *Generalized additive models*. CRC press.

Hawkins KA (2009) Is Chavez populist? Measuring populist discourse in comparative perspective. *Comparative Political Studies* 42(8): 1040–1067.

Hooghe L, Marks G and Wilson CJ (2002) Does left/right structure party positions on European integration? *Comparative Political Studies* 35(8): 965–989.

Huber RA and Schimpf CH (2017) On the distinct effects of left-wing and right-wing populism on democratic quality. *Politics and Governance* 5(4): 146–165.

Hunter W and Power TJ (2019) Bolsonaro and Brazil's illiberal backlash. *Journal of Democracy* 30(1): 68–82.

Karaveli H (2016) Erdogan's journey: Conservatism and authoritarianism in Turkey. *Foreign Affairs* 95(6): 121–130.

Katz RS (1980) *A theory of parties and electoral systems*. The John Hopkins University Press.

Kitschelt H and Rehm P (2014) Occupations as a site of political preference formation. *Comparative Political Studies* 47(12): 1670–1706.

Koopmans R (1996) Explaining the rise of racist and extreme right violence in Western Europe: Grievances or opportunities? *European Journal of Political Research* 30(2): 185–216.

Levitsky S and Roberts KM (2011) *The resurgence of the Latin American left*. JHU Press.

Levitsky S and Ziblatt D (2018) *How democracies die*. Broadway Books.

Linz JJ (1978) *Crisis, breakdown & reequilibration*. Johns Hopkins University Press.

Lührmann A, Dpont N, Higashijima M, Kavasoglu YB, Marquardt KL, Bernhard M, Dring H, Hicken A, Laebens M, Lindberg SI, Medzihorsky J et al. (2020a) Codebook Varieties of Party Identity and Organization (V-Party) .

Lührmann A and Lindberg SI (2019) A third wave of autocratization is here: what is new about it? *Democratization* 26(7): 1095–1113.

Lührmann A, Medzihorsky J, Hindle G and Lindberg SI (2020b) New global data on political parties: V-Party. *V-Dem Briefing Paper #9* : 1–4.

Maerz SF, Edgell AB, Wilson MC, Hellmeier S and Lindberg SI (2021) A framework for understanding regime transformation: Introducing the ERT dataset. *V-Dem Working Paper* 113.

Maerz SF and Schneider CQ (2019) Comparing public communication in democracies and autocracies: automated text analyses of speeches by heads of government. *Quality & Quantity* : 1–29.

Maerz SF and Schneider CQ (2021) Public discourse and autocratization: Infringing on autonomy, sabotaging accountability. *V-Dem Working Paper* (112).

Mainwaring S and Perez-Linan A (2013) *Democracies and dictatorships in Latin America: Emergence, survival, and fall*. Cambridge University Press.

Markowski R (2020) Plurality support for democratic decay: the 2019 Polish parliamentary election. *West European Politics* 43(7): 1513–1525.

Marks G, Hooghe L, Nelson M and Edwards E (2006) Party competition and European integration in the East and West: Different structure, same causality. *Comparative Political Studies* 39(2): 155–175.

Marquardt KL and Pemstein D (2018) IRT models for expert-coded panel data. *Political Analysis* 26(4): 431–456.

Marquardt KL, Pemstein D, Seim B and Wang Yt (2019) What makes experts reliable? expert reliability and the estimation of latent traits. *Research & Politics* 6(4): 1–8.

Marshall MG, Gurr TR and Jaggers K (2016) Polity IV project: Political regime characteristics and transitions, 1800–2015. *Center for Systemic Peace* 13.

McCoy J, Simonovits G and Littvay L (2020) Democratic hypocrisy: Polarized citizens support democracy-eroding behavior when their own party is in power. *APSA Preprints* .

Merkel W (2004) Embedded and defective democracies. *Democratization* 11(5): 33–58.

Mudde C and Kaltwasser CR (2013) Exclusionary vs. inclusionary populism: Comparing contemporary Europe and Latin America. *Government and Opposition* 48(2): 147–174.

Müller JW (2012) Militant democracy. *The Oxford handbook of comparative constitutional law* 1253: 1261.

Müller JW (2017) *What is populism?* Penguin UK.

Norris P and Inglehart R (2019) *Cultural backlash: Trump, Brexit, and authoritarian populism*. Cambridge University Press.

Pappas TS (2016) The specter haunting Europe: Distinguishing liberal democracy's challengers. *Journal of Democracy* 27(4): 22–36.

Pemstein D, Marquardt KL, Tzelgov E, Wang Yt, Medzihorsky J, Krusell J, Miri F and von Roemer J (2020) The V-Dem measurement model: Latent variable analysis for cross-national and cross-temporal expert-coded data. *V-Dem Working Paper* 21.

Plattner MF (2010) Democracys past and future: Populism, pluralism, and liberal democracy. *Journal of Democracy* 21(1): 8192.

Plattner MF (2015) Is democracy in decline? *Journal of Democracy* 26(1): 5–10.

Polk J and Rovny J (2017) Anti-elite/establishment rhetoric and party positioning on European integration. *Chinese Political Science Review* 2(3): 356–371.

Popper K (1945) *Open society and its enemies*. Routledge.

Przeworski A, Alvarez RM, Alvarez ME, Cheibub JA, Limongi F, Neto FPL et al. (2000) *Democracy and development: Political institutions and well-being in the world, 1950-1990*. Cambridge University Press.

Rohrschneider R and Whitefield S (2009) Understanding cleavages in party systems: issue position and issue salience in 13 post-communist democracies. *Comparative Political Studies* 42(2): 280–313.

Rooduijn M (2014) The nucleus of populism: In search of the lowest common denominator. *Government and Opposition* 49(4): 573–599.

Sartori G (1997) Understanding pluralism. *Journal of Democracy* 8(4): 58–69.

Schamis HE (2006) A "left turn" in Latin America? populism, socialism, and democratic institutions. *Journal of Democracy* 17(4): 20–34.

Schedler A (1996) Anti-political-establishment parties. *Party Politics* 2(3): 291–312.

Schedler A (2002) Elections without democracy: The menu of manipulation. *Journal of Democracy* 13(2): 36–50.

Singer M (2018) Delegating away democracy: How good representation and policy successes can undermine democratic legitimacy. *Comparative Political Studies* 51(13): 1754–1788.

Svolik MW (2008) Authoritarian reversals and democratic consolidation. *American Political Science Review* 102(1): 153–168.

Svolik MW (2015) Which democracies will last? Coups, incumbent takeovers, and the dynamic of democratic consolidation. *British Journal of Political Science* 45(4): 715–738.

Svolik MW (2020) When polarization trumps civic virtue: Partisan conflict and the subversion of democracy by incumbents. *Quarterly Journal of Political Science* 15(1): 3–31.

Taspinar O (2014) The end of the Turkish model. *Survival* 56(2): 49–64.

Varshney A (2019) Modi consolidates power: Electoral vibrancy, mounting liberal deficits. *Journal of Democracy* 30(4): 63–77.

Verbeek B and Zaslove A (2016) Italy: A case of mutating populism? *Democratization* 23(2): 304–323.

Volkens A, Krause W, Lehmann P, Matthiess T, Merz N, Regel S and Wessels B (2019) The manifesto data collection. Manifesto project (MRG/CMP/MARPOR). Version 2019b .

Weyland K (2013) Latin America's authoritarian drift: The threat from the populist left. *Journal of Democracy* 24(3): 18–32.

Wuttke A, Schimpf C and Schoen H (2020) When the whole is greater than the sum of its parts: On the conceptualization and measurement of populist attitudes and other multidimensional constructs. *American Political Science Review* 114(2): 356–374.

Zakaria F (1997) The rise of illiberal democracy. *Foreign Affairs* 76: 22.

Ziblatt D (2017) *Conservative Political Parties and the Birth of Modern Democracy in Europe*. Cambridge University Press.
